# Coculture of Marine *Streptomyces* sp. With *Bacillus* sp. Produces a New Piperazic Acid-Bearing Cyclic Peptide

**DOI:** 10.3389/fchem.2018.00498

**Published:** 2018-10-18

**Authors:** Daniel Shin, Woong Sub Byun, Kyuho Moon, Yun Kwon, Munhyung Bae, Soohyun Um, Sang Kook Lee, Dong-Chan Oh

**Affiliations:** Natural Products Research Institute, College of Pharmacy, Seoul National University, Seoul, South Korea

**Keywords:** marine microorganism, coculture, natural product, dentigerumycin, cyclic peptide

## Abstract

Microbial culture conditions in the laboratory, which conventionally involve the cultivation of one strain in one culture vessel, are vastly different from natural microbial environments. Even though perfectly mimicking natural microbial interactions is virtually impossible, the cocultivation of multiple microbial strains is a reasonable strategy to induce the production of secondary metabolites, which enables the discovery of new bioactive natural products. Our coculture of marine *Streptomyces* and *Bacillus* strains isolated together from an intertidal mudflat led to discover a new metabolite, dentigerumycin E (**1**). Dentigerumycin E was determined to be a new cyclic hexapeptide incorporating three piperazic acids, *N*-OH-Thr, *N*-OH-Gly, β-OH-Leu, and a pyran-bearing polyketide acyl chain mainly by analysis of its NMR and MS spectroscopic data. The putative PKS-NRPS biosynthetic gene cluster for dentigerumycin E was found in the *Streptomyces* strain, providing clear evidence that this cyclic peptide is produced by the *Streptomyces* strain. The absolute configuration of dentigerumycin E was established based on the advanced Marfey's method, ROESY NMR correlations, and analysis of the amino acid sequence of the ketoreductase domain in the biosynthetic gene cluster. In biological evaluation of dentigerumycin E (**1**) and its chemical derivatives [2-*N*,16-*N*-deoxydenteigerumycin E (**2**) and dentigerumycin methyl ester (**3**)], only dentigerumycin E exhibited antiproliferative and antimetastatic activities against human cancer cells, indicating that *N*-OH and carboxylic acid functional groups are essential for the biological activity.

## Introduction

Genomic analysis of microbes strongly suggests that microorganisms have more potential gene clusters that would allow them to produce more secondary metabolites than are currently known (Omura et al., 2001). Even if it is poorly understood, microorganisms are presumed to communicate with each other physically or/and chemically (Hogan and Kolter, 2002; Straight et al., 2006). Coculturing has been shown to alter individual cellular physiology and induce the production of microbial secondary metabolites that are genetically encoded but not produced under conventional laboratory culture conditions (Ueda and Beppu, 2017). In this context, coculturing different microbes to elicit the production of bioactive microbial compounds not previously observed when microbes are cultured independently could be a promising strategy to access microbial chemical diversity. A thorough literature search indicated that chemical studies of cocultures initially focused on mixed cultures of fungi and bacteria. The first new natural product from a coculture, pestalone, was discovered by cocultivation of the marine fungus *Pestalotia* sp. and the marine bacterium *Thalassospira* sp. (Cueto et al., 2001). A subsequent coculture experiment with the *Thalassospira* strain with the marine fungus *Libertella* sp. resulted in the production of new pimarane-type diterpenoids (Oh et al., 2005). In addition, the interaction between the fungus *Aspergillus fumigatus* and the bacterium *Sphingomonas* afforded glionitrin A (Park et al., 2009). Relatively recently, cocultures of two different bacterial strains have also successfully contributed to the discovery of new bioactive natural products. Cocultivating various *Streptomyces* strains with *Tsukamurella pulmonis* elicited the production of new butanolides (Hoshino et al., 2015). Ecologically relevant cocultures of marine invertebrate-associated *Rhodococcus* and *Micromonospora* strains produced the antibiotic bis-nitroglycosylated anthracycline (Adnani et al., 2017). As part of our efforts to discover new bioactive molecules from marine bacteria, we adopted the coculture strategy. Chemical analysis of a coculture of two marine bacterial strains isolated together from an intertidal mudflat in Wando, Republic of Korea, showed the induction of the formation of a bacterial metabolite. This result prompted us to scale up the coculture and subsequently characterize the metabolite. Here, we report the production, structure elucidation, putative biosynthetic gene cluster (BGC), and biological activity of the new bacterial metabolite, dentigerumycin E (**1**).

## Materials and methods

### General experimental procedures

Specific rotations were obtained using a JASCO P-2000 polarimeter with a 1-cm cell at 25°C. UV spectra were obtained using an Applied Photophysics Chirascan™-plus spectrometer with a 1-cm quartz cell at 25°C. IR spectral data were obtained using a JASCO FT/IR-4200 spectrometer. NMR spectra were recorded on an 800 MHz Bunker Avance III HD spectrometer with a 5-mm TCI cryoprobe and a Bunker Avance 600 MHz spectrometer at the National Center for Inter-university Research Facilities (NCIRF). LC-MS and low-resolution electrospray ionization mass spectroscopic (LR-ESI-MS) data were obtained using an Agilent Technologies 1200 series high performance liquid chromatography (HPLC) coupled with an Agilent Technologies 6130 quadrupole MS. High-resolution fast atom bombardment MS (HR-FAB-MS) data were obtained using a JEOL JMS-700 high-resolution MS at NCIRF.

### Bacterial isolation

A mud sample was collected from the intertidal mudflat in Wando (34°18′55.5″N 126°45′21.8″E), Republic of Korea in September 2014. For bacterial isolation from the sample, 1 g of the mud sample was diluted in 10 mL of sterilized artificial seawater, and the mixture was spread onto A4 medium, actinomycete isolation agar medium, starch casein medium, chitin-based medium, Czapek-Dox agar medium, Bennet's agar medium, YPM agar medium, YPG agar medium, and K agar medium (all agar media were made with artificial seawater and 100 mg/L cycloheximide). These isolation agar plates were incubated at 25°C for 3 weeks. The actinobacterial strain JB5 and the *Bacillus* strain GN1 were isolated on the same plate together from actinomycete isolation agar medium. The strain JB5 was identified as *Streptomyces* sp. (99% identical to *Streptomyces albogriseolus* strain B24) on the basis of 16S rRNA gene sequence analysis (GenBank accession No. MH656702). The strain GN1 was identified as *Bacillus* sp. (99% identical to *Bacillus cereus*) by 16S rRNA gene sequence analysis (GenBank accession No. MH656703).

### Coculture experiment

*Streptomyces* sp. strain JB5 and various strains in different phyla were cultivated separately in 50 mL of YEME liquid medium (4 g of yeast extract, 10 g of malt extract, and 4 g of glucose as a 40% solution in 1 L of artificial seawater) in a 125-mL Erlenmeyer flask. After 4 days of cultivation in a rotary shaker at 200 rpm at 30°C, equal volumes of the liquid cultures of JB5 and other strains were mixed (10 mL to 10 mL) and inoculated into a 500-mL baffled Erlenmeyer flask containing 200 mL of YEME liquid medium. Mixed strains were cocultivated over 8 days in a rotary shaker at 200 rpm at 30°C, and the chemical profiles of the cocultures were monitored by LC-MS every 2 days. The strains cocultivated with JB5 were *Bacillus* sp. (GN1), *Streptomyces* sp. (SD53; isolated from the gut of *Bombyx mori*), *Paenibacillus* sp. (CC2; isolated from the gut of *Meloe proscarabaeus*), *Brevibacillus* sp. (PTH23; isolated from the excreta of *Onthophagus lenzii*), *Streptomyces* sp. (UTZ13; isolated from the *Nicrophorus concolor* parasitic mites), *Mycobacterium* sp. (Myc06; isolated from the vegetable mold formed by *Oligochaeta*), *Hafnia* sp. (CF1; isolated from the *Meloe proscarabaeus*), and *Bacillus* sp. (HR1; isolated from the gut of *Pseudopyrochroa rufula*).

### Genome sequencing and analysis

The genome of the JB5 strain was constructed de novo using Pacbio sequencing data. Genome sequencing of the JB5 strain was performed using the PacBio RS II by Chunlab, Inc. (Seoul, Republic of Korea), and sequencing data were assembled with PacBio SMRT Analysis 2.3.0 using the HGAP2 protocol (Pacific Biosciences, USA). Nucleotide sequences with 122.76-fold coverage of the *Streptomyces* sp. JB5 genome (~7.72 Mbp) were generated. Gene prediction was performed using Prodigal 2.6.2, and sequences were annotated with ChunLab's in-house pipeline with EggNOG 4.5, Swissprot, KEGG, and SEED as references. The dentigerumycin E BGC was identified using antiSMASH (Medema et al., 2011; Weber et al., 2015).

### Production and extraction of dentigerumycin E

JB5 and GN1 strains were cultivated separately in 50 mL of YEME liquid medium in 125-mL Erlenmeyer flasks. After 4 days of cultivation in a rotary shaker at 200 rpm at 30°C, 10 mL of the JB5 culture and 1 mL of the GN1 culture (based on a serial dilution, approximately 4.9 × 10^7^ cells were estimated to be in 1 mL of *Bacillus* sp. GN1 culture) were inoculated together into a 500-mL baffled Erlenmeyer flask containing 200 mL of YEME liquid medium. The *Streptomyces* sp. JB5 and *Bacillus* sp. GN1 were cocultivated for 6 days in a rotary shaker at 200 rpm at 30°C. A total of 120 L of the coculture was extracted twice with 180 L of EtOAc by using a separation funnel. The EtOAc layer was separated from the aqueous phase, and the residual water in the organic layer was removed by adding anhydrous sodium sulfate.

### Isolation of dentigerumycin E

The crude extract was filtered through a 25HP045AN syringe filter unit and directly injected onto a semipreparative reversed-phase HPLC column (YMC-Pack ODS-A, 250 × 10 mm, C_18_, 5 μm) and was separated with a gradient solvent system (flow rate: 2 mL/min; UV detection: 210 nm; 35% to 50% CH_3_CN/H_2_O with 0.1% formic acid over 50 min). Dentigerumycin E (**1**) eluted at a retention time of 27 min and was further purified by semipreparative HPLC using gradient solvent conditions (column: YMC-Pack ODS-A, 250 × 10 mm, C_18_, 5 μm, flow rate: 2 mL/min; UV detection: 230 nm; 66 to 88% gradient MeOH/H_2_O with 0.1% formic acid over 20 min). Pure dentigerumycin E (34 mg) was obtained at a retention time of 38 min under the final purification conditions.

*Dentigerumycin E* (**1**): white, amorphous powder; ${\left[\alpha \right]}_{D}^{25}$ = −3.1 (c 0.01, MeOH); UV (MeOH) λ_max_ (log ε) 204 (3.36) nm; IR (neat) ν_max_ 3401, 2929, 1741, 1644, 1507, 1445, 1248, 1196 cm^−1^; HR-FAB-MS [M+Na]^+^ *m/z* 936.4290 (calcd for C_39_H_63_N_9_O_16_Na, 936.4290); For ^1^H and ^13^C NMR spectral data, Table 1.

**Table 1:** ^1^H and ^13^C NMR spectral data of dentigerumycin E (**1**) in pyridine-*d*_5_^*a*^.

**Position**	**δ_C_**	**Type**	**δ_H_**	**mult (*J* in Hz)**	**Position**	**δ_C_**	**Type**	**δ_H_**	**mult (*J* in Hz)**
1	170.6	C			20a	21.3	CH_2_	1.68	m
2	66.9	CH	4.19	d (10.0)	20b			1.37	m
2-N-OH			n.d.		21a	47.4	CH_2_	3.11	m
3	68.8	CH	6.0	m	21b			2.95	br. d (15.5)
3-OH			n.d.		21-NH			5.69	br. d (13.0)
4	19.0	CH_3_	1.89	d (6.5)	22	169.3	C		
5	169.6	C			23	57.0	CH	5.83	dd (10.0, 10.0)
6	52.7	CH	5.44	dd (5.5, 1.5)	23-NH			9.13	d (10.0)
7a	24.4	CH_2_	2.25	br. d (14.0)	24	77.8	CH	5.95	dd (10.0, 1.0)
7b			1.62	m	25	30.6	CH	2.46	m
8a	22.0	CH_2_	1.21	m	26	15.5	CH_3_	1.35	d (7.0)
8b			1.06	br. d (13.0)	27	20.4	CH_3_	1.25	d (7.0)
9a	47.3	CH_2_	3.03	br. d (13.0)	28	178.2	C		
9b			2.69	m	29	77.8	C		
9-NH			4.90	br. d (13.0)	29-OH			6.97	s
10	176.3	C			30	100.0	C		
11	43.7	CH	6.55	br. d (6.5)	30-OH			7.03	s
12a	25.2	CH_2_	1.84	br. d (13.0)	31a	28.3	CH_2_	2.26	m
12b			1.68	m	31b			2.06	m
13a	20.4	CH_2_	2.02	m	32a	26.0	CH_2_	2.10	m
13b			1.26	m	32b			2.05	m
14a	47.6	CH_2_	2.93	br. d (14.5)	33	36.9	CH	2.17	m
14b			2.59	m	34	75.2	CH	4.03	m
14-NH			5.59	dd (12.5, 1.0)	35	22.7	CH_3_	1.75	s
15	167.4	C			36a	38.0	CH_2_	2.62	dd (15.0, 4.5)
16a	52.6	CH_2_	5.21	d (17.5)	36b			2.28	dd (15.0, 8.5)
16b			4.82	d (17.5)	37	175.2	C		
16-N-OH			n.d.		37-OH			n.d.	
17	174.1	C			38a	25.7	CH_2_	1.72	m
18	48.7	CH	6.22	br. d (6.5)	38b			1.45	m
19a	25.8	CH_2_	2.42	br. d (13.5)	39	9.7	CH_3_	0.98	t (7.5)
19b			1.92	m					

a *H and ^13^C NMR data were recorded at 800 and 200 MHz, respectively*.

### Reduction of *N*-OHs in dentigerumycin E

Dentigerumycin E (**1**, 10 mg) was dissolved in 4 mL of THF (tetrahydrofuran). Aqueous ammonium acetate (2 mL, 4.5 M) and 1 mL of 12% TiCl_3_ solution in 20-30 wt. % HCl were added to the solution. The mixture was stirred at room temperature for 2 h, and the product was extracted with 20 mL of EtOAc. The organic layer was concentrated *in vacuo*, and the reaction product (2-*N*,16-*N*-deoxydentigerumycin E, **2**) was purified by reversed-phase HPLC (YMC-Pack ODS-A, 250 × 10 mm, C_18_, 5 μm) with an isocratic solvent system (flow rate: 2 mL/min; UV detection: 210 nm; 36% CH_3_CN/H_2_O with 0.1% formic acid). 2-*N*,16-*N*-deoxydentigerumycin E (**2**, 3.1 mg) eluted at 38.2 min, and its molecular formula was confirmed as C_39_H_63_N_9_O_14_ by HR-FAB mass spectroscopic analysis. The ^1^H and ^13^C NMR chemical shifts of 2-*N*,16-*N*-deoxydentigerumycin E (**2**) were assigned based on ^1^H NMR, HSQC, COSY, HMBC, ROESY, and TOCSY analyses.

*2-N,16-N-deoxydentigerumycin E* (**2**): white, amorphous powder; ${\left[\alpha \right]}_{D}^{25}$ = −2.6 (c 0.01, MeOH); UV (MeOH) λ_max_ (log ε) 200 (3.31) nm; IR (neat) ν_max_ 3385, 2935, 1745, 1640, 1511, 1314, 1246, 1201 cm^−1^; HR-FAB-MS [M+Na]^+^ *m/z* 904.4399 (calcd for C_39_H_63_N_9_O_16_Na, 904.4392); For ^1^H and ^13^C NMR spectral data, Table S1.

### Application of advanced Marfey's method

Dentigerumycin E (**1**, 0.6 mg) was hydrolyzed in 0.5 mL of 6 N HCl at 115°C for 1 h with stirring, and the reaction was quenched by cooling in an ice bath for 3 min. The HCl was evaporated *in vacuo*. Then, 0.5 mL of water was added to the dry material and dried *in vacuo* three times to remove the residual HCl. The hydrolysate was lyophilized for 24 h to ensure complete removal of the acid. The hydrolysate containing free amino acids was divided into two vials. Each hydrolysate was dissolved in 200 μL of 1 N NaHCO_3_, and 100 μL of l-FDAA (1-fluoro-2,4,dinitrophenyl-5-l-alanine amide) or d-FDAA in acetone (10 mg/mL) was added to each vial. Both vials were incubated at 80°C for 3 min, and 100 μL of 2 N HCl was added to neutralize the reactions. Each reaction mixture was diluted with 300 μL of 50% CH_3_CN/H_2_O solution, and 20-μL aliquots of each reaction product were analyzed by LC-MS using a gradient solvent system (flow rate: 0.7 mL/min; UV detection: 340 nm; 10 to 60% CH_3_CN/H_2_O with 0.1% formic acid over 40 min) with a reversed-phase column (Phenomenex Luna C_18_(2), 100 × 4.6 mm, C_18_, 5 μm). To determine the stereochemistry of *N*-OH Thr, the reduction product (**2**, 1.5 mg) was hydrolyzed with 6 N HCl at 115°C for 1 h with stirring. The hydrolysate of **2** was derivatized with Marfey reagents (l-FDAA and d-FDAA), and the FDAA-adducts were analyzed by LC-MS in the same manner as described above.

### GITC (2,3,4,6-tetra-*O*-acetyl-β-d-glucopyranosyl isothiocyanate) derivatization

Authentic standards of l-*allo*-Thr and l-Thr (0.5 mg) along with the hydrolysate of the reduction product (**2**) were dissolved in 200 μL of water in 4-mL vials. Then, 200 μL of 6% trimethylamine and 1% GITC (2,3,4,6-tetra-*O*-acetyl-β-d-glucopyranosyl isothiocyanate) in acetone were added to each vial. The reaction mixtures were stirred at 25°C for 15 min, and the derivatization was quenched by adding 100 μL of 5% acetic acid. Aliquots of each reaction mixture (40 μL) were analyzed by LC-MS under gradient HPLC conditions (flow rate: 0.7 mL/min; UV detection: 254 nm; 10 to 100% CH_3_CN/H_2_O with 0.1% formic acid over 50 min, column: Phenomenex Luna C_18_(2), 100 × 4.6 mm, 5 μm).

### Methylation of dentigerumycin E

Dentigerumycin E (**1**, 10 mg) was dissolved in 3 mL of anhydrous MeOH. AcCl (2.5 μL) was added to the solution, and the mixture was stirred at room temperature for 9 h with LC-MS monitoring of the reaction progress. The reaction mixture was concentrated *in vacuo* and purified by reversed-phase HPLC (YMC-Pack ODS-A, 250 × 10 mm, C_18_, 5 μm) with an isocratic solvent system (flow rate: 2 mL/min; UV detection: 210 nm; 38% CH_3_CN/H_2_O with 0.1% formic acid). Dentigerumycin E methyl ester (**3**, 1.5 mg) eluted at 41.5 min under these HPLC conditions. Its molecular formula was confirmed as C_40_H_65_N_9_O_16_ by HR-FAB mass spectroscopic analysis. The ^1^H and ^13^C NMR chemical shifts of dentigerumycin E methyl ester (**3**) were assigned by ^1^H NMR, HSQC, COSY, HMBC, ROESY, and TOCSY spectral analyses.

*Dentigerumycin E* methyl ester (**3**): white, amorphous powder; ${\left[\alpha \right]}_{D}^{25}$ = −1.3 (c 0.012, MeOH); UV (MeOH) λ_max_ (log ε) 205 (3.65) nm; IR (neat) ν_max_ 3263, 2935, 1738, 1643, 1508, 1442, 1355, 1247, 1195 cm^−1^; HR-FAB-MS [M+Na]^+^ *m/z* 950.4442 (calcd for C_40_H_65_N_9_O_16_Na, 950.4447); For ^1^H and ^13^C NMR spectral data, Table S1.

### Cell culture

Human cancer cells (A549, HCT116, MDA-MB-231, SK-HEP-1) and human breast epithelial cell (MCF-10A) were obtained from the American Type Culture Collection (Manassas, VA, USA) and a stomach cancer cell (SNU-638) was obtained from the Korean Cell Line Bank (Seoul, Korea). The cells were cultured in an appropriate medium (Dulbecco's modified Eagle's medium for MDA-MB-231 and SK-HEP-1; Roswell Park Memorial Institute 1640 for A549, HCT116 and SNU-638 cells; Dulbecco's modified Eagle's medium: Nutrient Mixture F-12 for MCF-10A) supplemented with antibiotics-antimycotics (PSF: 100 units/mL sodium penicillin G, 100 μg/mL streptomycin, and 250 ng/mL amphotericin B) and 10% fetal bovine serum (FBS) in an incubator containing 5% CO_2_ at 37°C. All reagents were purchased from Gibco® Invitrogen Corp. (Grand Island, NY, USA).

### Cell proliferation assay

Cell proliferation was measured by a sulforhodamine B (SRB) assay. Briefly, cells were seeded in 96-well plates and incubated for 30 min (for zero day controls) or treated with **1**-**3** for the indicated times. After incubation, the cells were fixed, dried and stained with 0.4% SRB in 1% acetic acid. Unbound dye was removed by washing, and the stained cells were suspended in 10 mM Tris (pH 10.0). The absorbance was measured at 515 nm, and the cell proliferation was determined. IC_50_ values were calculated by nonlinear regression analysis using TableCurve 2D v5.01 software (Systant Software Inc., Richmond, CA, USA). All reagents were purchased from Sigma-Aldrich.

### Wound healing assay

MDA-MB-231 cells were grown to 80–90% confluence in a 6-well plate. A confluent monolayer of MDA-MB-231 cells was artificially wounded with a 200 μL pipette tip, and the detached cells were washed with phosphate-buffered saline (PBS, Invitrogen Corp.) and then incubated with 1% FBS in medium containing various concentrations of **1**–**3** for 24 h. The wounds were photographed at 0 and 24 h using an inverted microscope (Olympus, Tokyo, Japan). The wound area was quantified using ImageJ Software (National Institutes of Health) and presented as wound healing (%) relative to the area of the wound at 0 h.

### Cell invasion assay

Twenty-four-well transwell membrane inserts 6.5-mm in diameter with 8 μm pores (Corning, Tewksbury, MA, USA) were coated with 10 μL of type I collagen (0.5 mg/mL, BD Biosciences, San Diego, CA, USA) and 20 μL of a 1:20 mixture of Matrigel (BD Biosciences)/PBS. After treatment with the test compounds for 24 h, MDA-MB-231 cells were harvested, resuspended in serum-free medium, and plated (1 × 10^6^ cells/chamber) in the upper chamber of the Matrigel-coated transwell insert. Medium containing 30% FBS was used as the chemoattractant in the lower chambers. After 24 h of incubation, the cells that had invaded the outer surface of the lower chambers were fixed and stained using a Diff-Quik Staining Kit (Sysmex, Kobe, Japan) and imaged. Representative images from 3 separate experiments are shown, and the number of invaded cells was counted in 5 randomly selected microscopic fields (200 × magnification) (Kim et al., 2018).

## Results and discussion

### Coculture experiments for the production of dentigerumycin E

**Figure 1:**
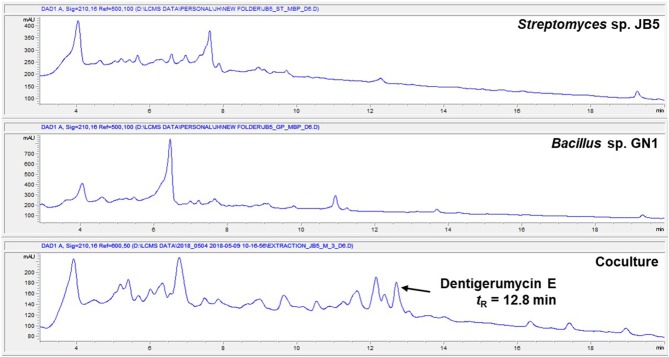
HPLC profiles of the single culture of *Streptomyces* sp. JB5, the single culture of *Bacillus* sp. GN1, and their coculture.

The marine *Streptomyces* sp. JB5, isolated from an intertidal mudflat in Wando, Republic of Korea, was cocultivated with the marine *Bacillus* sp. GN1, which was associated with the *Streptomyces* strain JB5 (Figure S1). The production of secondary metabolites was monitored by LC-MS every 2 days. the coculture with marine *Bacillus* sp. GN1, which was isolated from the intertidal mudflat along with *Streptomyces* sp. JB5, displayed a significantly different chemical profile compared with those of the pure cultures of the strains JB5 and GN1. In particular, on the sixth day cocultivation, LC-MS analysis of the coculture of JB5 and GN1 showed a newly produced metabolite at a retention time of 12.8 min that was not virtually detected in the cultures of the individual strains (Figure 1, Figure S2). The observed compound, later identified as a new cyclic depsipeptide, dentigerumycin E (**1**), showed a UV absorption maximum at 204 nm and a molecular ion [M+H]^+^ at *m*/*z* 914. To optimize the production of dentigerumycin E, the two bacterial strains were mixed in various ratios. After 4 days of individual cultivation, the two pure cultures of JB5 and GN1 were mixed in ratios of 1:1, 2:1, 5:1, and 10:1. LC-MS analysis of the cocultures indicated that a 10:1 ratio of JB5/GN1 provides the best yield of dentigerumycin E (**1**).

The *Streptomyces* sp. JB5 was also cocultivated with phylogenetically diverse but ecologically irrelevant bacterial strains including *Bacillus* sp. HR1, *Paenibacillus* sp. CC2, *Brevibacillus* sp. PTH23, *Streptomyces* sp. SD53, *Streptomyces* sp. UTZ13, *Hafnia* sp. CF1, and *Mycobacterium* sp. Myc06. Most of the cocultures of *Streptomyces* sp. JB5 with these bacterial strains did not show the production of dentigerumycin E or the induction of other metabolites, the coculture with *Bacillus* sp. HR1, which is phylogenetically close to *Bacillus* sp. GN1, produced dentigerumycin E (Figure S3). Although the mechanism triggering the biosynthesis of dentigerumycin E by *Bacillus* strains remains unclear, these coculture experiment results implied that *Bacillus* strains, which are most closely related to *B. cereus*, possibly have common ability to induce the production of dentigerumycin E from *Streptomyces* sp. JB5.

### Structure elucidation of dentigerumycin E

Dentigerumycin E (**1**) was isolated as an amorphous white power with a molecular formula of C_39_H_63_N_9_O_16_ based on HR-FAB-MS analysis (obsd. [M+Na]^+^ at *m*/*z* 936.4290, calcd. 936.4290). The molecular formula indicated that this molecule possesses 13 degrees of unsaturation. The ^13^C NMR spectrum of **1** indicated eight carbonyl carbons (δ_C_: 178.2, 176.3, 175.2, 174.1, 170.6, 169.4, 169.3, and 167.4), one dioxygenated carbon (δ_C_: 100.0), 13 N/O-bound carbons (δ_C_: 77.8, 77.8, 75.2, 68.8, 66.9, 57.0, 52.7, 52.6, 48.7, 47.6, 47.4, 47.3, and 43.7), and 18 alkyl carbons (δ_C_: 38.0 ~ 9.7) (Table 1). The ^1^H and HSQC NMR spectra of **1** indicated six exchangeable protons (δ_H_: 9.13, 7.03, 6.97, 5.69, 5.59, and 4.90), three oxygenated methines (δ_C_/δ_H_: 77.8/5.95, 75.2/4.03, and 68.8/6.00), five methines at the α-positions of amino acids (δ_C_/δ_H_: 66.9/4.19, 57.0/5.83, 52.7/5.44, 48.7/6.22, and 43.7/6.55), four nitrogenous methylenes (δ_C_/δ_H_: 52.6–5.21 and 4.82, 47.6/2.93 and 2.59, 47.4/3.11 and 2.95, and 47.3/3.03 and 2.69), two alkyl methines, 10 alkyl methylenes, one singlet methyl (δ_C_/δ_H_: 22.71.75), three doublet methyls (δ_C_/δ_H_: 20.4/1.25, 19.0/1.89, and 15.5/1.35), and one triplet methyl (δ_C_/δ_H_: 9.7/0.98) (Table 1).

**Figure 2:**
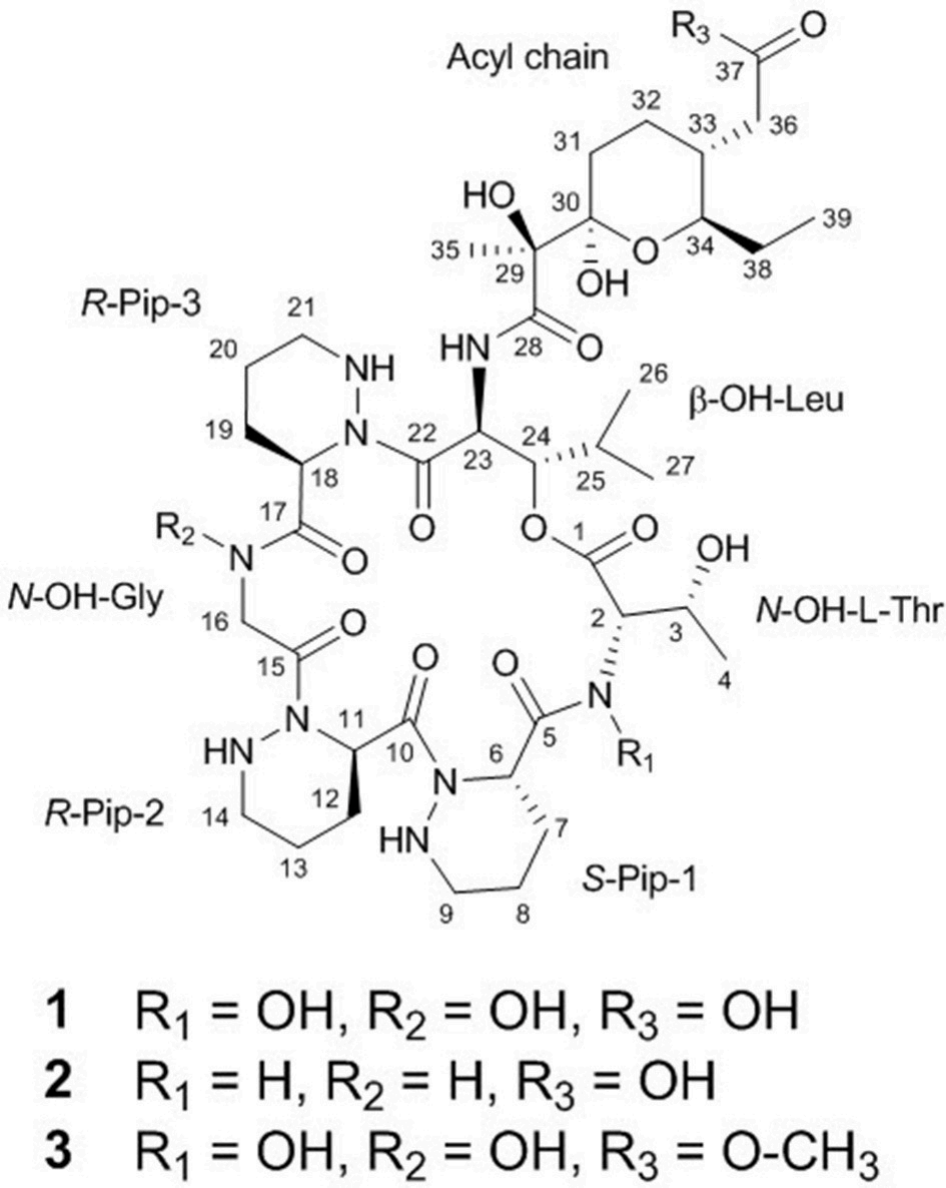
Structures of dentigerumycin E (**1**) and its derivatives (**2** and **3**).

The COSY, TOCSY, and HMBC NMR spectra indicated that dentigerumycin E (**1**) possesses six unusual amino acid residues and one polyketide-derived substructure (Figure 2, Figures S4–S10). First, an array of COSY/TOCSY correlations constructed a spin system from H-6 to 9-NH. The HMBC correlation from H-6 to the C-5 carbonyl carbon and from 9-NH to C-6 demonstrated that this spin system includes a piperazic acid (Pip-1). Two additional piperazic acid units (Pip-2 and Pip-3) were similarly assigned based on the corresponding COSY, TOCSY, and HMBC correlations. Further analysis of COSY and TOCSY spectroscopic data revealed another discrete spin system from 23-NH to two doublet methyl groups (H_3_-26 and H_3_-27). The α-amino proton H-23 displayed an HMBC correlation with the carbonyl carbon at C-22, indicative of a β-hydroxy leucine (β-OH-Leu). In addition, a *N*-hydroxy threonine unit (*N*-OH-Thr) was proposed based on the COSY/TOCSY correlations among H-2, H-3, and H_3_-4. An independent α-amino methylene signal correlated with the C-15 carbonyl carbon in the HMBC NMR spectrum indicated the presence of *N*-hydroxy glycine (*N*-OH-Gly).

**Figure 3:**
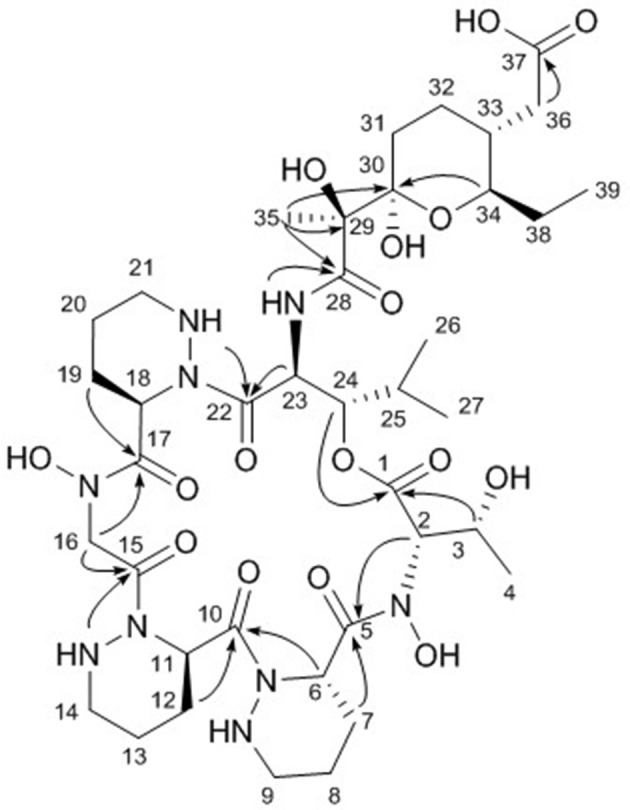
Key HMBC correlations (arrows) in dentigerumycin E (**1**).

The final structural fragment was assigned as a pyran-bearing polyketide-derived moiety. A terminal triplet methyl group (C-39) was connected to C-38 by the H_3_-39/H_2_-38 COSY correlation. The 3-bond ^1^H-^1^H couplings between H_2_-38 and H-34 placed the C-39-C-38 ethyl group next to C-34. Subsequently, the COSY and TOCSY correlations among H-34, H-33, H_2_-32, and H_2_-31 constructed an alkyl chain spin system from C-39 to C-31. H_2_-36 displayed a COSY coupling with H-33, indicating C-33 was a branch point. The 3-bond ^1^H-^13^C correlations from H_2_-36 to the C-37 carbonyl carbon connected C-37 to C-36. The alkyl chain was further extended from C-31 to C-30, a dioxygenated carbon, based on the H_2_-31/C-30 HMBC correlation. A singlet methyl signal (H_3_-35) exhibited strong HMBC correlations to C-30, C-29, and C-28, which finally constructed a C_9_ backbone chain with a C_2_ branch and a branch methyl group. 29-OH and 30-OH, which were visible in the ^1^H NMR spectrum, were assigned at C-29 and C-30, respectively, based on their HMBC correlations. Further analysis of the HMBC spectrum revealed the presence of a pyran ring by the H-34/C-30 3-bond correlation. Therefore, the polyketide-derived partial structure was determined to be a C_12_ alkyl moiety bearing a pyran ring and a branching C_2_ chain and a branch methyl group (Figure 3).

The six amino acids and the polyketide-derived acyl chain were assembled by analysis of the HMBC spectrum. *N*-OH-Thr was located adjacent to Pip-1 based on the H-2/C-5 and H_2_-7/C-5 ^1^H-^13^C correlations. The HMBC correlations from H-6 and H-12 to C-10 connected Pip-1 and Pip-2. The sequence from Pip-2 to *N*-OH-Gly was established by heteronuclear couplings from 14-NH and H_2_-16 to C-15. H_2_-16 also displayed an HMBC correlation to C-17. The connectivity between *N*-OH-Gly and Pip-3 was supported by the H_2_-19/C-17 HMBC coupling. β-OH-Leu was located next to Pip-3 based on the 3-bond correlations from 21-NH and H-23 to C-22. The polyketide-derived moiety was connected to β-OH-Leu by the 28-NH/C-28 HMBC correlation. Therefore, the sequence of the partial structures of **1** was determined to be *N*-OH-Thr-Pip-1-Pip-2-*N*-OH-Gly-Pip-3-β-OH-Leu-polyketide-derived acyl chain (Figure 3).

The carbonyl groups explained 8 of the 13 double bond equivalents, indicating that dentigerumycin E (**1**) must be a pentacyclic compound. The three piperazic acids and pyran ring accounted for another four of the double bond equivalents. Thus, dentigerumycin E must possess an additional ring. The 3-bond HMBC correlation between H-24 and C-1 constructed a macrocycle (Figure 3). After elucidating most of the structure of **1**, the C-37 carbonyl carbon was proposed to be a carboxylic acid based on the molecular formula, which allowed the planar structure of dentigerumycin E (**1**) to be proposed as shown.

The uncertainty in the planar structure of **1** due to the invisibility of the *N*-OH and carboxylic OH moieties in the ^1^H NMR spectrum was further clarified by utilizing chemical reactions and NMR spectroscopic analyses of the products. To confirm the presence of two *N*-OH groups, the *N*-OHs were converted to NHs using TiCl_3_ (Pennings et al., 1983). The structure of the reduction product (2-*N*,16-*N*-deoxydentigerumycin E, **2**) (Figure 2) was elucidated based on the ^1^H NMR, COSY, HMBC, HSQC, TOCSY, and ROESY spectra (Table S1, Figures S11–S16) and HRMS data, confirming the presence of the *N*-OH groups of *N*-OH-Thr and *N*-OH-Gly in dentigerumycin E (**1**). On the other hand, the only HMBC correlation to the C-37 carbonyl carbon (δ_C_ 175.2) was from H_2_-36, not fully proving the proposed carboxylic acid functionality. To validate the presence of the carboxylic acid group in the acyl side chain, we performed an AcCl-mediated methylation and confirmed the structure of the methylated product (dentigerumycin E methyl ester, **3**) (Figure 2) by ^1^H NMR, COSY, HMBC, HSQC, TOCSY, and ROESY (Table S1, Figures S17–S22) as well as HRMS analysis. The protons of the methoxy group displayed a clear HMBC correlation to the carbonyl carbon in **3**, confirming the carboxylic acid functionality in **1**. Therefore, the planar structure of dentigerumycin E (**1**) was unequivocally elucidated.

**Figure 4:**
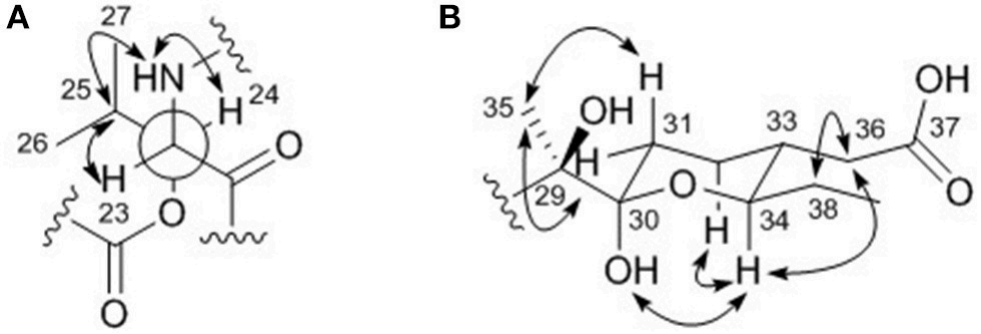
Key ROESY correlations (double headed arrows) for determining the relative configurations of **(A)** the β-hydroxy leucine and **(B)** the acyl chain in dentigerumycin E (**1**).

The relative configuration of the two congruent stereogenic centers in β-hydroxy leucine were established through *J*-based configuration analysis (Figure 4A). The large ^1^H-^1^H coupling between H-23 and H-24 (*J*_H23H24_ = 10.0 Hz) indicated that these two protons have *anti*-relationship. Further ROESY correlations allowed the assignment of 23S^*^ and 24S^*^ (Matsumori et al., 1999). The relative configuration of the pyran ring in the acyl side chain was determined by ROESY correlations (Figure 4B).

To establish the absolute configuration of dentigerumycin E (**1**), acid hydrolysis was performed using 6 N HCl at 115°C for 1 h. The hydrolysate was then derivatized with Marfey's reagents (l-FDAA and d-FDAA) and analyzed by LC-MS (Fujii et al., 1997). The l-FDAA and d-FDAA derivatives of β-hydroxy leucine were detected at retention times of 15.2 min and 18.2 min, determining its l(*S*)-configuration at the α-amino position (Figure S23). Based on the advanced Marfey method and the previously determined relative stereochemistries, the absolute configuration of the chiral centers in the β-hydroxy leucine were determined to be 23*S* and 24*S*. The l-FDAA and d-FDAA derivatives of the three piperazic acid units eluted at retention times of 11.9 min and 14.1 min in a ratio of 2:1. Based on Marfey analysis of synthetic (*S*)- and (*R*)-piperazic acids, the l-FDAA derivative of piperazic acid elutes faster than the d-FDAA derivative when piperazic acid has an *R*-configuration (Oh et al., 2009). Therefore, dentigerumycin E contains two *R*-Pip and one *S*-Pip units.

Because the free *N*-OH-Thr does not react with Marfey's reagents, 2-*N*,16-*N*-deoxydentigerumycin E (**2**), which possesses Thr converted from *N*-OH-Thr, was hydrolyzed and derivatized with Marfey's reagents. In the LC-MS analysis of the derivatives, the l-FDAA and d-FDAA derivatives of threonine eluted at 18.2 min and 21.4 min, respectively, indicating the α-position of threonine was in the l (*S*) configuration (Figure S23). Due to the presence of the additional stereogenic center at the β-carbon in threonine, GITC (2,3,4,6-tetra-*O*-acetyl-β-d-glucopyranosyl isothiocyanate) derivatization was performed (Hess et al., 2004). The retention times of the GITC derivatives of **2** and authentic standards of l-*allo*-Thr and l-Thr were compared. The GITC derivatives of l-*allo*-Thr, l-Thr, and Thr in **2** eluted at retention times of 14.7 min, 15.1 min, and 15.1 min, respectively (Figure S24). Therefore, the *N*-hydroxy threonine of dentigerumycin E (**1**) was determined to be *N*-OH-l-Thr, and thus the absolute configurations of its stereogenic centers were determined to be 2*S* and 3*R*.

### Analysis of the putative biosynthetic pathway of dentigerumycin E for stereochemical assignment

**Figure 5:**
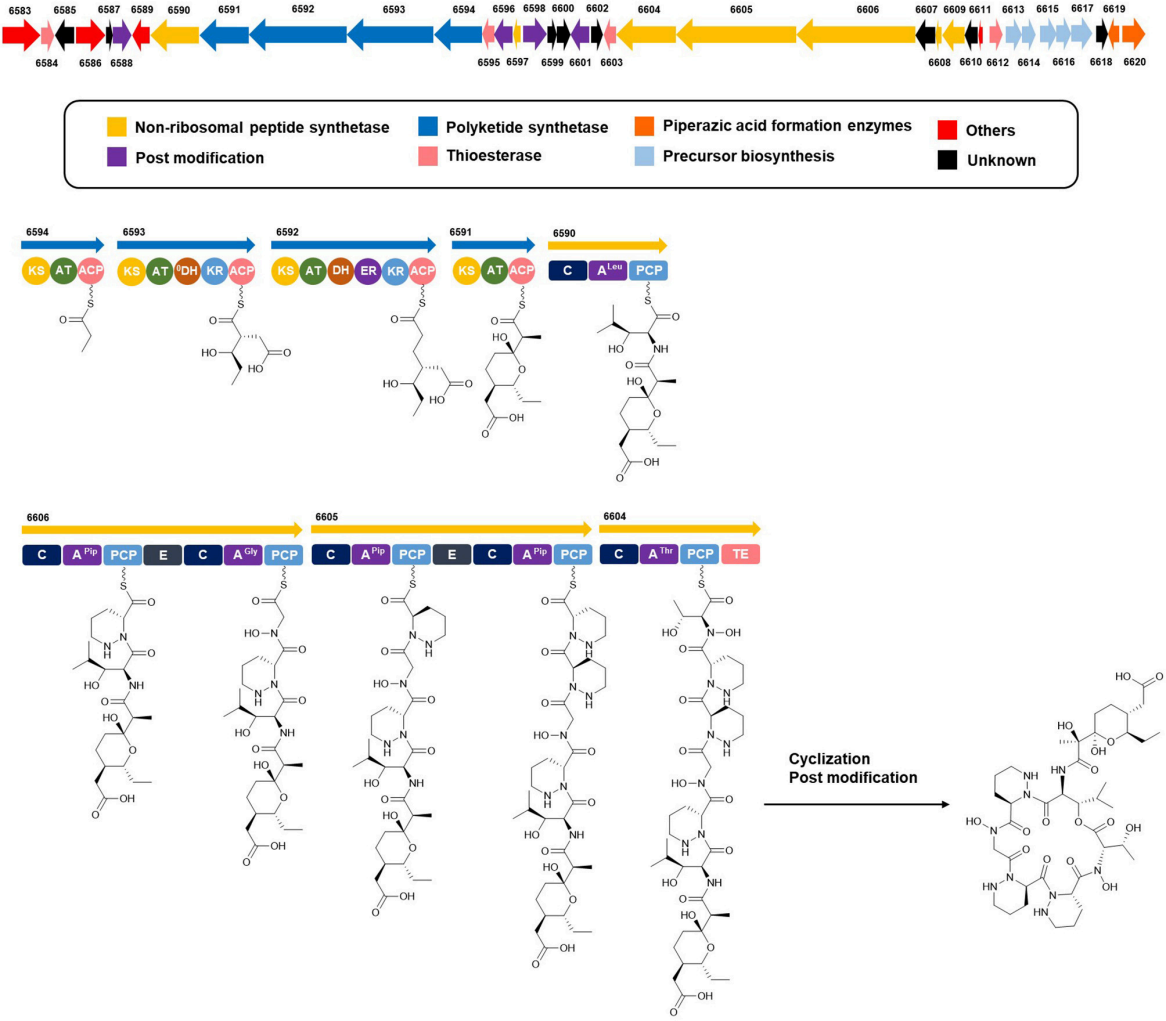
Putative biosynthetic gene cluster and proposed biosynthetic pathway of dentigerumycin E (**1**).

Complete assignment of the sequence of the two *R*- and one *S*-piperazic acid moieties was accomplished through analysis of the putative biosynthetic gene cluster (BGC) (Table S2). Full genome sequencing of *Streptomyces* sp. JB5 allowed the identification of the BGC of dentigerumycin E (**1**). Even though its origin was reasonably inferred as *Streptomyces* sp. JB5 based on previous reports of dentigerumycin class compounds (Oh et al., 2009; Wyche et al., 2017), identifying the BGC added clearer evidence of its *Streptomyces* origin because sometimes the same class of compounds can be discovered together from phylogenetically diverse bacteria (Blodgett et al., 2010). As predicted based on the structure, the BGC was composed of a multimodular hybrid polyketide synthase (PKS) and a nonribosomal peptide synthetase (NRPS) (Figure 5). The feature of NRPS involving six modules in the four open reading frames (ORFs 6590, 6606, 6605, and 6604) showed high degree similarity to that of dentigerumycins B-D (Wyche et al., 2017). Based on the antiSMASH analysis, these modules produce a cyclic peptide with the sequence Leu-Pip-3-Gly-Pip-2-Pip-1-Thr, which is consistent with the structure elucidated by spectroscopic analysis. Epimerase domains, which determine the absolute configurations of the amino acid residues in NRPS, were incorporated in the modules for Pip-3 and Pip-2 but not Pip-1. The 2:1 ratio of *R*- and *S*-piperazic acids determined by advanced Marfey's analysis (*vide supra*) established that the absolute configuration of the three piperazic acids is 6*S*, 11*R*, and 18*R* (*S*-Pip-1, *R*-Pip-2, and *R*-Pip-3) (Figure 5).

**Figure 6:**
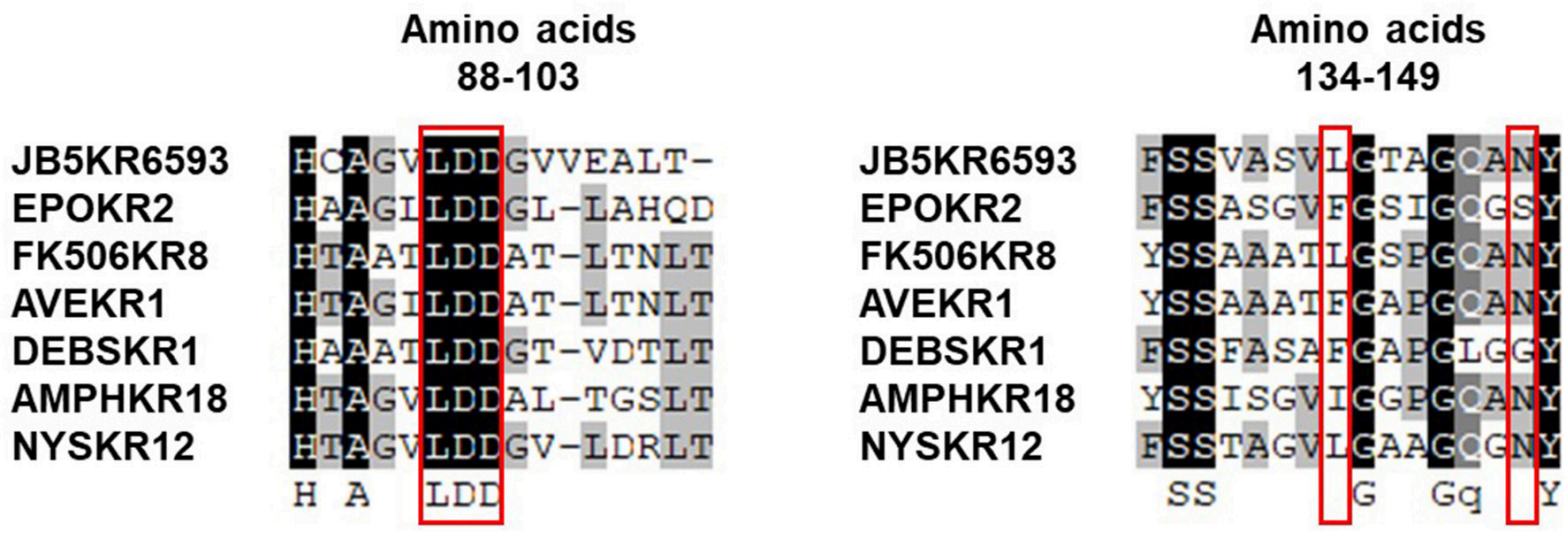
Sequence alignment of KR domains in the biosynthetic gene cluster of dentigerumycin E (JB5KR6593) and various other PKSs reported by Caffrey (2003).

The absolute configuration of the acyl side chain was also assigned through analysis of the BGC. Within the BGC of dentigerumycin E, four type I PKS modules (ORFs 6594, 6593, 6592, and 6591) were involved in the biosynthesis of the C_12_ acyl side chain (Figure 5). Both module 6594 and 6591 are composed of a ketosynthase (KS), an acyltransferase (AT), and an acyl carrier protein (ACP). Because the KS of 6594 was identified as KS_Q_ with the active site cysteine (C) being replaced by glutamine (Q) (Kuhstoss et al., 1996), 6594 was identified as the loading module of dentigerumycin E BGC (Figure S25). Module 6593 involves KS, AT, dehydratase (DH), ketoreductase (KR), and ACP. In modular polyketide synthase (PKS), certain amino acid motifs in the KR domain are correlated with the stereochemistry of the hydroxyl groups in the product (Caffrey, 2003; Reid et al., 2003). KR domains lead to A-type alcohol stereochemistry (3*S* when C-2 has higher priority than C-4 and 3*R* when C-4 has higher priority than C-2) if amino acid residue 141 is tryptophan (W) and B-type alcohol stereochemistry (3*R* when C-2 has higher priority than C-4 and 3*S* when C-4 has higher priority than C-2) if an LDD motif is present in the region between 88 and 103. In addition, B-type KR domains typically contain proline (P) or/and asparagine (N) at residue 144 and 148, respectively. During the reaction with PKS, it was hypothesized that the tryptophan motif guides the polyketide into the active site of A-type KRs, while the LDD motif guides polyketides into the active site of B-type KRs (Zheng et al., 2010). The KR domain of module 6593, which determines the absolute configuration of C-34 hydroxy group, was aligned with other KR domains reported by Caffrey (2003), and the residues were compared. Based on the LDD motif, the KR domain of module 6593 was identified as B-type, indicating the product should have a 34*R* configuration (Figure 6). *R*-configuration is also supported by the evidence that residue 141 was not a tryptophan and the presence of asparagine at residue 148. Based on the relative configuration previously determined by ROESY correlations, the absolute configuration of the stereogenic centers in the acyl side chain should be 29*S*, 30*R*, 33*R*, and 34*R* (Figure 2).

On the other hand, because the hydroxy group at C-34 was preserved rather than eliminated, the DH domain in module 6593 is assumed to be nonfunctional, similar to what is seen with polyoxypeptin A (Du et al., 2014), which has a BGC that closely resembles that of the dentigerumycin class of compounds. Although this DH domain of dentigerumycin E has all three key amino acids residues of the conserved motif HxxxGxxxxP, the motif is not always universally conserved (Joshi and Smith, 1993). In addition, the two tyrosine (Y) residues in the GYxYGPxF motif were altered to phenylalanine (F) and histidine (H), respectively. Such a significant alteration in these conserved motifs could cause the domain to be nonfunctional (Keatinge-Clay, 2008), and these alterations in the GYxYGPxF motif could explain the nonfunctional DH in module 6593. (Figure S26).

### Biological activity of dentigerumycin E and its derivatives

**Figure 7:**
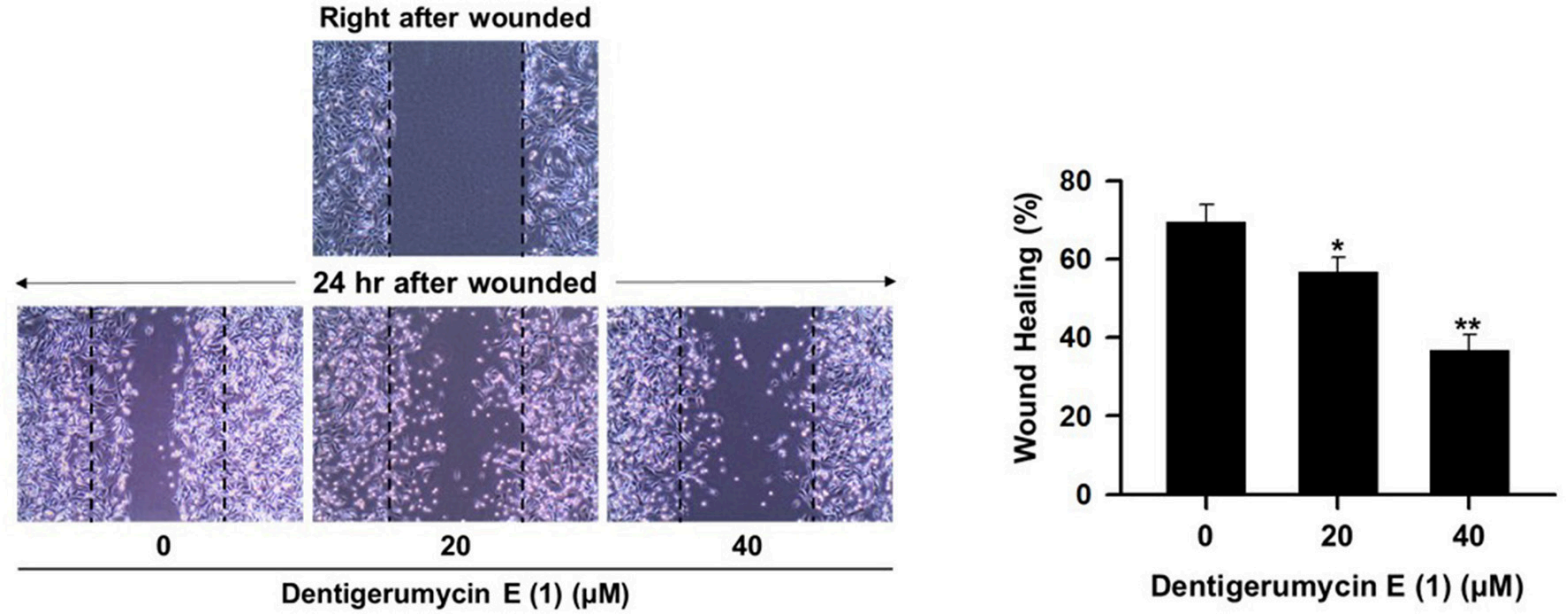
Dentigerumycin E (**1**) suppresses cell migration of the metastatic human breast cancer cell line (MDA-MB-231) in the wound healing assay. The data are represented as the means ± SD from three independent experiments: *p < 0.05, **p < 0.01.

**Figure 8:**
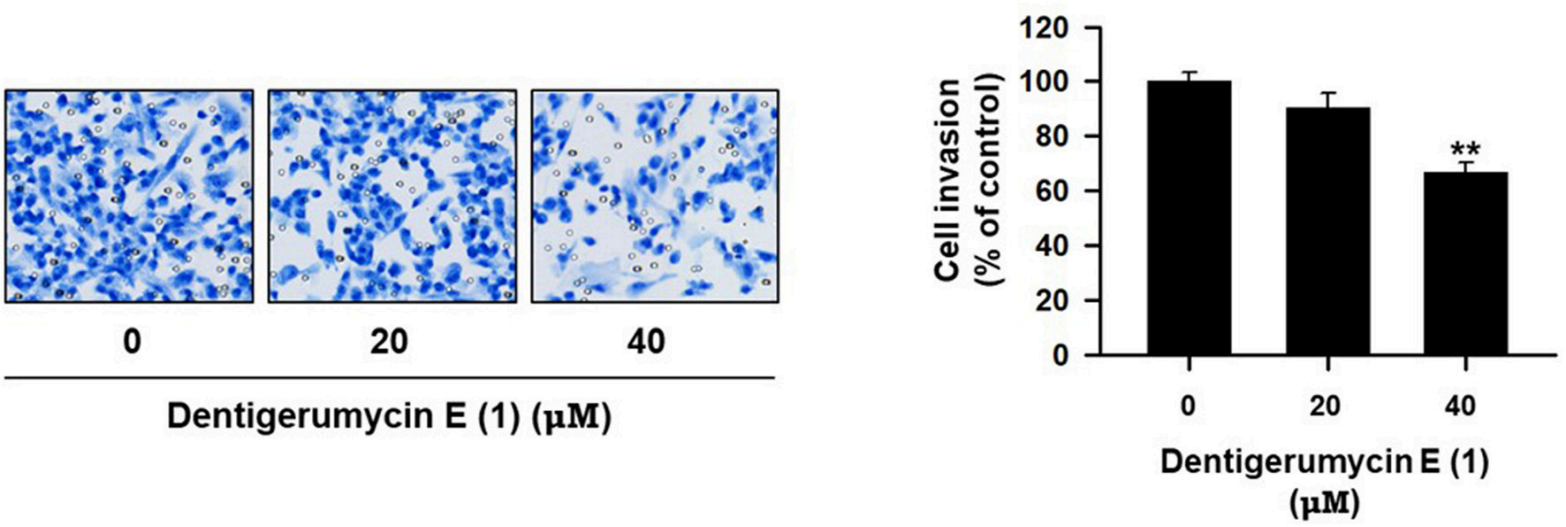
Dentigerumycin E suppresses cell invasion of the human breast cancer cell line (MDA-MB-231) in the cell invasion assay. The data are represented as the means ± SD from three independent experiments: **p < 0.01.

To explore the biological activities of dentigerumycin E (**1**) and its derivatives (**2** and **3**), cytotoxicity was evaluated against various human cancer cells. Dentigerumycin E (**1**) showed moderate cytotoxicity against the tested cancer cell lines including A549 (lung cancer), HCT116 (colorectal cancer), MDA-MB-231 (breast cancer), SK-HEP-1 (liver cancer), and SNU638 (stomach cancer) whereas 2*N*,16*N*-deoxydentigerumycin E (**2**) and dentigerumycin E methyl ester (**3**) did not inhibit the proliferation of these cancer cell lines (Table S3). To determine the cancer cell-specific cytotoxicity, cytotoxicity of **1**-**3** against a normal human breast epithelial cell line (MCF-10A) was also evaluated. As shown in Table S3, in comparison to cancer cell lines, dentigerumycin E (**1**) did not demonstrated a significant cytotoxicity against normal epithelial cells with IC_50_ value of over 50 μM. Furthermore, the antimetastatic activity of dentigerumycin E was evaluated with metastatic breast cancer cells (MDA-MB-231) by wound healing and cell invasion assays. For each assay, cells were treated with 20 μM or 40 μM dentigerumycin E for 24 h. Compared to the vehicle-treated group, dentigerumycin E inhibited cell migration in the wound healing assay by 20 and 48% at 20 and 40 μM, respectively (Figure 7). In the cell invasion assay, dentigerumycin E exhibited inhibitory activity by 10 and 34% at 20 and 40 μM, respectively (Figure 8). These results indicated the antimetastatic potential of dentigerumycin E (**1**) against breast cancer cells. However, 2*N*,16*N*-deoxydentigerumycin E (**2**) and dentigerumycin E methyl ester (**3**) displayed no significant activity in the wound healing and cell invasion assays (Figures S27, S28), suggesting that 2-*N*-OH, 16-*N*-OH, and 37-OH (carboxylic acid) in **1** are essential for its antiproliferative and antimetastatic activities.

## Conclusion

A new cyclic peptide, dentigerumycin E (**1**), was discovered from a coculture of marine *Streptomyces* sp. JB5 and *Bacillus* sp. GN1 strains isolated from an intertidal mudflat. Cocultivating *Streptomyces* sp. JB5 and a *Bacillus* strain (either GN1 or HR1), most closely related *B. cereus*, was required because dentigerumycin E was virtually detectable only in cocultures of these bacterial strains, not in single cultures of these bacteria. Dentigerumycin E was determined to be a new member of the dentigerumycin nonribosomal peptide class with three piperazic acid units and an acyl chain of PKS origin (Oh et al., 2009). Dentigerumycin E (**1**) is most similar to dentigerumycin B, previously reported from the *Pseudonocardia* symbiont of a fungus-growing ant (Wyche et al., 2017), but the *N*-hydroxy threonine moiety in **1** has a different configuration from that in dentigerumycin B (l-Thr for dentigerumycin E whereas l-*allo*-Thr for dentigerumycin B). Moreover, the polyketide-derived acyl chain of **1** has a carboxylic acid group in the branched C_2_ chain, which has not been reported in the other dentigerumycin-type compounds. Our biological evaluation of the antiproliferative and antimetastatic activities revealed that the 2-*N*-OH, 16-*N*-OH, and 37-OH groups are essential for the activities of dentigerumycin E. Genomic analysis of the *Streptomyces* sp. JB5 strain allowed the identification of the putative BGC of dentigerumycin E (**1**) and thus confirmed that **1** was produced by the *Streptomyces* strain. In conjunction with chemical derivatizations, comprehensive analysis of the biosynthetic modules allowed the absolute configurations of the piperazic acid residues and PKS-derived acyl chain to be proposed. Induction of dentigerumycin E production by a couple of phylogenetically close *Bacillus* strains raised an interesting question about the mechanism activating the biosynthetic gene cluster for dentigerumycin E in *Streptomyces* sp. JB5, which might lead a more comprehensive mechanism study. The discovery of dentigerumycin E (**1**) in the marine bacterial coculture highlights that marine microorganisms are prolific chemical sources of bioactive natural products and that the coculture strategy could be a promising method for exploring hidden microbial chemical diversity.

## Author contributions

DS, WB, KM, YK, MB, SU, SL, and D-CO designed the experiments. YK and MB collected intertidal mud samples. KM isolated the bacterial strains. DS performed the coculture experiments, chemical experiments, and analyzed the data. WB and SL performed the bioassay. DS, WB, KM, SU, SL, and D-CO wrote the manuscript.

### Conflict of interest statement

The authors declare that the research was conducted in the absence of any commercial or financial relationships that could be construed as a potential conflict of interest.

## References

[B1] Adnani N. Chevrette M. G. Adibhatla S. N. Zhang F. Yu Q. Braun D. R.. (2017). Coculture of marine invertebrate-associated bacteria and interdisciplinary technologies enable biosynthesis and discovery of a new antibiotic, keyicin. ACS Chem. Biol. 12, 3093–3102. 10.1021/acschembio.7b00688 29121465 PMC5973552

[B2] Blodgett J. A. V. Oh D.-C. Cao S. Currie C. R. Kolter R. Clardy J. (2010). Common biosynthesis origins of polycyclic tetramate macrolactams from phylogenetically diverse bacteria. Proc. Natl. Acd. Sci. U.S.A. 107, 11692–11697. 10.1073/pnas.1001513107 PMC2900643 20547882

[B3] Caffrey P. (2003). Conserved amino acid residues correlating with ketoreductase stereospecificity in modular polyketide synthases. ChemBioChem 4, 649–662. 10.1002/cbic.200300581 12851937

[B4] Cueto M. Jensen P. R. Kauffman C. Fenical W. Lobkovsky E. Clardy J. (2001). Pestalone, a new antibiotic produced by a marine fungus in response to bacterial challenge. J. Nat. Prod. 64, 1444–1446. 10.1021/np0102713 11720529

[B5] Du Y. Wang Y. Huang T. Tao M. Deng Z. Lin S. (2014). Identification and characterization of the biosynthetic gene cluster of polyoxypeptin A, a potent apoptosis inducer. BMC Microbiol. 14:30. 10.1186/1471-2180-14-30 24506891 PMC3943440

[B6] Fujii K. Ikai Y. Oka H. Suzuki M. Harada K. (1997). A nonempirical method using LC/MS for determination of the absolute configuration of constituent amino acids in a peptide: combination of Marfey's method with mass spectrometry and its practical application. Anal. Chem. 69, 5146–5151. 10.1021/ac970289b

[B7] Hess S. Gustafson K. R. Milanowski D. J. Alvira E. Lipton M. A. Pannell L. K. (2004). Chirality determination of unusual amino acids using precolumn derivatization and liquid chromatography-electrospray ionization mass spectrometry. J. Chromatogr. A 1035, 211–219. 10.1016/j.chroma.2004.02.068 15124814 PMC1484300

[B8] Hogan D. A. Kolter R. (2002). *Pseudomonas-Candida* interactions: an ecological role for virulence factors. Science 296, 2229–2232. 10.1126/science.1070784 12077418

[B9] Hoshino S. Wakimoto T. Onaka H. Abe I. (2015). Chojalactones A–C, cytotoxic butanolides isolated from *Streptomyces* sp. cultivated with mycolic acid containing bacterium. Org. Lett. 17, 1501–1504. 10.1021/acs.orglett.5b00385 25742189

[B10] Joshi A. K. Smith S. (1993). Construction, expression, and characterization of a mutated animal fatty acid synthase deficient in the dehydrase function. J. Biol. Chem. 268, 22508–22513. 8226759

[B11] Keatinge-Clay A. T. (2008). Crystal structure of the erythromycin polyketide synthase dehydratase. J. Mol. Biol. 384, 941–953. 10.1016/j.jmb.2008.09.084 18952099 PMC3496180

[B12] Kim W. K. Byun W. S. Chung H.-J. Oh J. Park H. J. Choi J. S.. (2018). Esculetin suppresses tumor growth and metastasis by targeting Axin2/E-cadherin axis in colorectal cancer. Biochem. Pharmacol. 152, 71–83. 10.1016/j.bcp.2018.03.009 29534875

[B13] Kuhstoss S. Huber M. Turner J. R. Paschal J. W. Rao R. N. (1996). Production of a novel polyketide through the construction of a hybrid polyketide synthase. Gene 183, 231–236. 10.1016/S0378-1119(96)00565-3 8996112

[B14] Matsumori N. Kaneno D. Murata M. Nakamura H. Tachibana K. (1999). Stereochemical determination of acyclic structures based on carbon-proton spin-coupling constants. A method of configuration analysis for natural products. J. Org. Chem. 64, 866–876. 10.1021/jo981810k 11674159

[B15] Medema M. H. Blin K. Cimermancic P. de Jager V. Zakrzewski P. Fischbach M. A.. (2011). antiSMASH: rapid identification, annotation and analysis of secondary metabolite biosynthesis gene clusters in bacterial and fungal genome sequences. Nucleic Acids Res. 39, W339–W346. 10.1093/nar/gkr466 21672958 PMC3125804

[B16] Oh D.-C. Jensen P. R. Kauffman C. A. Fenical W. (2005). Libertellenones A–D: induction of cytotoxic diterpenoid biosynthesis by marine microbial competition. Bioorg. Med. Chem. 13, 5267–5273. 10.1016/j.bmc.2005.05.068 15993608

[B17] Oh D.-C. Poulsen M. Currie C. R. Clardy J. (2009). Dentigerumycin: a bacterial mediator of an ant-fungus symbiosis. Nat. Chem. Biol. 5, 391–393. 10.1038/nchembio.159 19330011 PMC2748230

[B18] Omura S. Ikeda H. Ishikawa J. Hanamoto A. Takahashi C. Shinose Y.. (2001). Genome sequence of an industrial microorganism *Streptomyces avermitilis*: deducing the ability of producing secondary metabolites. Proc. Natl. Acad. Sci. U.S.A. 98, 12215–12220. 10.1073/pnas.211433198 11572948 PMC59794

[B19] Park H. B. Kwon H. C. Lee C. H. Yang H. O. (2009). Glionitrin A an antibiotic-antitumor metabolite derived from competitive interaction between abandoned mine microbes. J. Nat. Prod. 72, 248–252. 10.1021/np800606e 19159274

[B20] Pennings M. L. M. Reinhoudt D. N. Harkema S. van Hummel G. J. (1983). Chemistry of four-membered cyclic nitrones. 4. Reaction with electrophilic reagents and conversion into β-lactam derivatives. J. Org. Chem. 48, 486–491. 10.1021/jo00152a015

[B21] Reid R. Piagentini M. Rodriguez E. Ashley G. Viswanathan N. Carney J.. (2003). A model of structure and catalysis for ketoreductase domains in modular polyketide synthases. Biochemistry 42, 72–79. 10.1021/bi0268706 12515540

[B22] Straight P. D. Willey J. M. Kolter R. (2006). Interactions between *Streptomyces coelicolor* and *Bacillus subtilis*: role of surfactants in raising aerial structures. J. Bacteriol. 188, 4918–4925. 10.1128/JB.00162-06 16788200 PMC1483000

[B23] Ueda K. Beppu T. (2017). Antibiotics in microbial coculture. J. Antibiot. 70, 361–365. 10.1038/ja.2016.127 27756913

[B24] Weber T. Blin K. Duddela S. Krug D. Kim H. U. Bruccoleri R.. (2015). antiSMASH 3.0-a comprehensive resource for the genome mining of biosynthetic gene clusters. Nucleic Acids Res. 43, W237–W243. 10.1093/nar/gkv437 25948579 PMC4489286

[B25] Wyche T. P. Ruzzini A. C. Beemelmanns C. Kim K. H. Klassen J. L. Cao S.. (2017). Linear peptides are the major products of a biosynthetic pathway that encodes for cyclic depsipeptides. Org. Lett. 19, 1772–1775. 10.1021/acs.orglett.7b00545 28326787 PMC6013059

[B26] Zheng J. Taylor C. A. Piasecki S. K. Keatinge-Clay A. T. (2010). Structural and functional analysis of A-type ketoreductase from amphotericin modular polyketide synthase. Structure 18, 913–922. 10.1016/j.str.2010.04.015 20696392

