# Supplementary material for: Coculture of Marine Streptomyces sp. With Bacillus sp. Produces a New Piperazic Acid-Bearing Cyclic Peptide
Source: Front Chem. 2018 Oct 18;6:498. doi: 10.3389/fchem.2018.00498 (PMC6201156; doi:10.3389/fchem.2018.00498)

Supplementary Material

Coculture of Marine *Streptomyces* sp. with *Bacillus* sp. Produces a New Piperazic Acid-Bearing Cyclic Peptide

Daniel Shin, Woong Sub Byun, Kyuho Moon, Yun Kwon, Munhyung Bae, Soohyun Um, Sang Kook Lee, Dong-Chan Oh*

*** Correspondence:** Prof. Dong-Chan Oh: dongchanoh@snu.ac.kr

Table of Contents

Figure S1. *Streptomyces* sp. strain JB5 (a) and *Bacillus* sp. strain GN1 (b).

Figure S2. LC/MS profiles of the single culture of *Streptomyces* sp. JB5, the single culture of *Bacillus* sp. GN1, and their coculture.

Figure S3. Chemical profiles of cocultures.

Figure S4. ^{1}H NMR spectrum (800 MHz) of dentigerumycin E (**1**) in pyridine-d_5.

Figure S5. ^{13}C NMR spectrum (200 MHz) of dentigerumycin E (**1**) in pyridine-d_5.

Figure S6. COSY NMR spectrum (800 MHz) of dentigerumycin E (**1**) in pyridine-d_5.

Figure S7. HSQC NMR spectrum (800 MHz) of dentigerumycin E (**1**) in pyridine-d_5.

Figure S8. HMBC NMR spectrum (800 MHz) of dentigerumycin E (**1**) in pyridine-d_5.

Figure S9. ROESY NMR spectrum (800 MHz) of dentigerumycin E (**1**) in pyridine-d_5.

Figure S10. TOCSY NMR spectrum (800 MHz) of dentigerumycin E (**1**) in pyridine-d_5.

Figure S11. ^{1}H NMR spectrum (800 MHz) of 2-*N*,16-*N*-deoxydentigerumycin E (**2**) in pyridine-d_5.

Figure S12. COSY NMR spectrum (800 MHz) of 2-*N*,16-*N*-deoxydentigerumycin E (**2**) in pyridine-d_5.

Figure S13. HSQC NMR spectrum (800 MHz) of 2-*N*,16-*N*-deoxydentigerumycin E (**2**) in pyridine-d_5.

Figure S14. HMBC NMR spectrum (800 MHz) of 2-*N*,16-*N*-deoxydentigerumycin E, **2**) in pyridine-d_5.

Figure S15. ROESY NMR spectrum (800 MHz) of 2-*N*,16-*N*-deoxydentigerumycin E (**2**) in pyridine-d_5.

Figure S16. TOCSY NMR spectrum (800 MHz) of 2-*N*,16-*N*-deoxydentigerumycin E (**2**) in pyridine-d_5.

Figure S17. ^{1}H NMR spectrum (600 MHz) of dentigerumycin E methyl ester (**3**) in pyridine-d_5.

Figure S18. COSY NMR spectrum (600 MHz) of dentigerumycin E methyl ester (**3**) in pyridine-d_5.

Figure S19. HSQC NMR spectrum (600 MHz) of dentigerumycin E methyl ester (**3**) in pyridine-d_5.

Figure S20. HMBC NMR spectrum (600 MHz) of dentigerumycin E methyl ester (**3**) in pyridine-d_5.

Figure S21. ROESY NMR spectrum (600 MHz) of dentigerumycin E methyl ester (**3**) in pyridine-d_5.

Figure S22. TOCSY NMR spectrum (600 MHz) of dentigerumycin E methyl ester (**3**) in pyridine-d_5.

Table S1. NMR spectral data for 2-*N*,16-*N*-deoxydentigerumycin (**2**) and dentigerumycin E methyl ester (**3**) in pyridine-d_5.

Figure S23. LC/MS chromatogram of (a) L- and (b) D-FDAA derivatives of **1**, and (c) L- and D-FDAA derivatives of threonine in **2**.

Figure S24. LC/MS chromatogram of GITC of (a) **2**, (b) authentic L-Thr, and (c) authentic L-*allo*-Thr.

Table S2. Alignment of the homologous proteins from the dentigerumycin E biosynthetic gene cluster.

Figure S25. Sequence alignment of KS domains from BGC of dentigerumycin E.

Figure S26. Sequence alignment of DH domains from BGC of dentigerumycin E and other PKSs.

Table S3. Antiproliferative activities of **1-3** against various human cancer cell lines and normal breast epithelial cells.

Figure S27. Wound healing assay of 2-*N*,16-*N*-deoxydentigerumycin E (**2**) and dentigerumycin E methyl ester (**3**).

Figure S28. Transwell cell invasion assay 2-*N*,16-*N*-deoxydentigerumycin E (**2**) and dentigerumycin E methyl ester (**3**).

.

Figure S1. *Streptomyces* sp. strain JB5 (a) and *Bacillus* sp. strain GN1 (b).

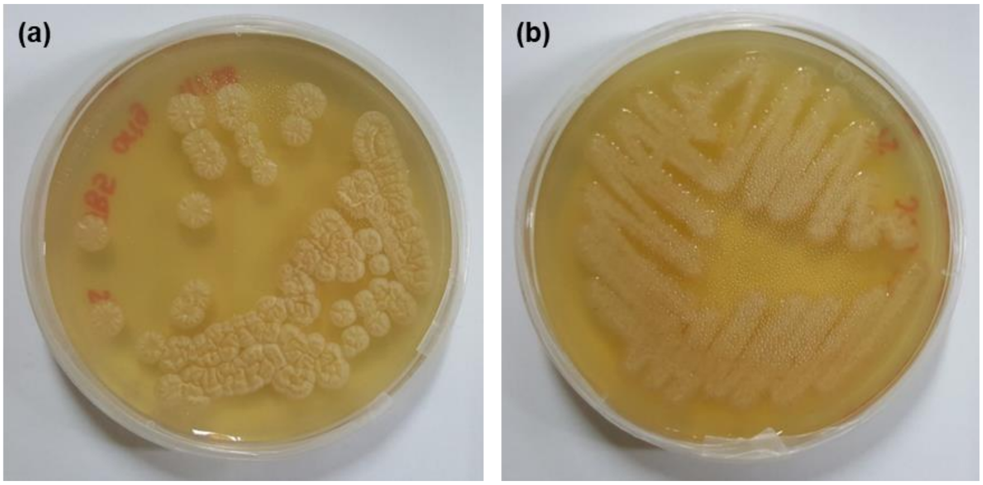

Figure S2. LC/MS profiles of the single culture of *Streptomyces* sp. JB5, the single culture of *Bacillus* sp. GN1, and their coculture.

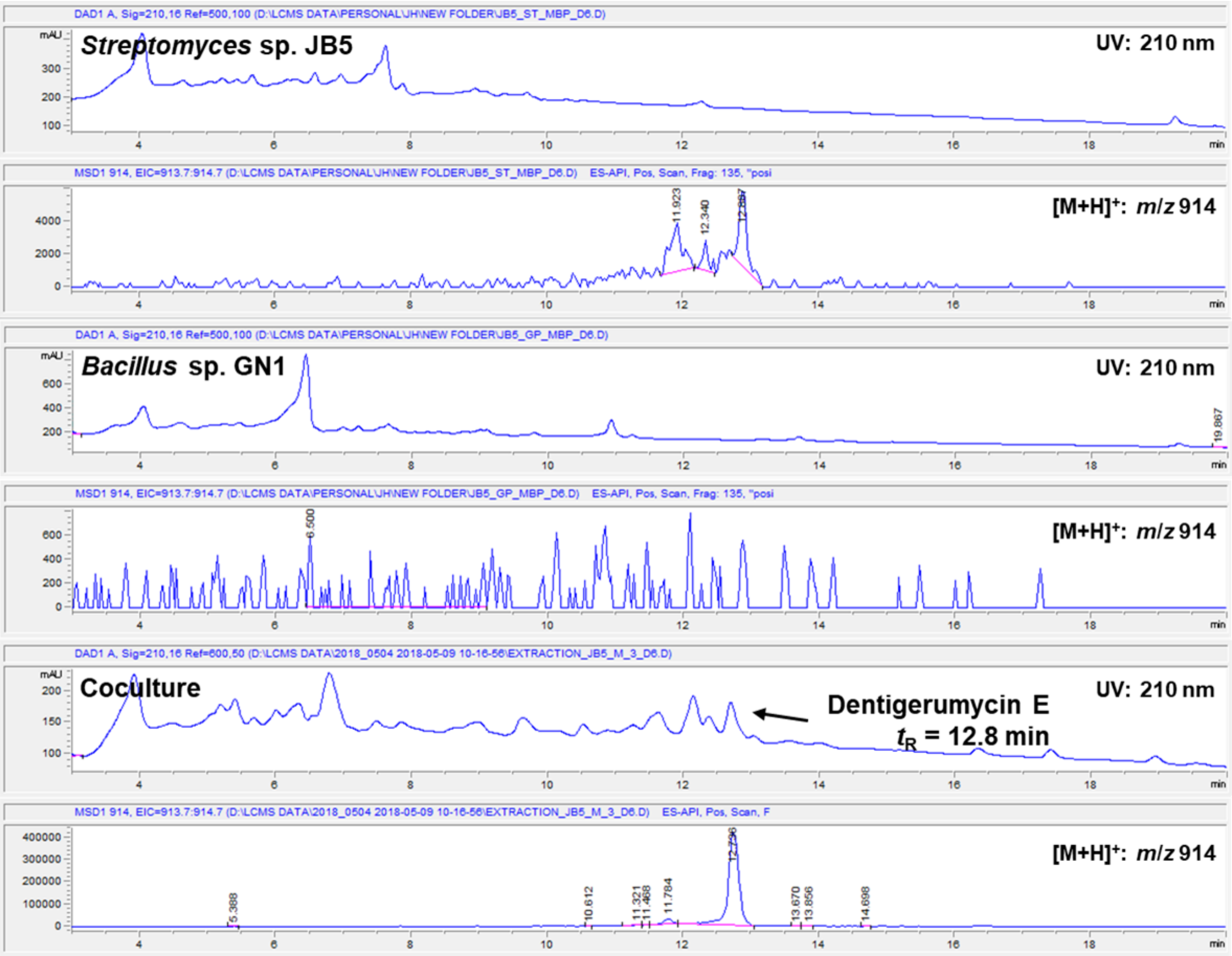

Figure S3. Chemical profiles of cocultures of *Streptomyces* sp. JB5 with (a) *Bacillus* sp. GN1, (b) *Streptomyces* sp. SD53, (c) *Paenibacillus* sp. CC2, (d) *Brevibacillus* sp. PTH23, (e) *Streptomyces* sp. UTZ13, (f) *Hafnia* sp. CF1, (g) *Mycobacterium* sp. Myc06, and (h) *Bacillus* sp. HR1.

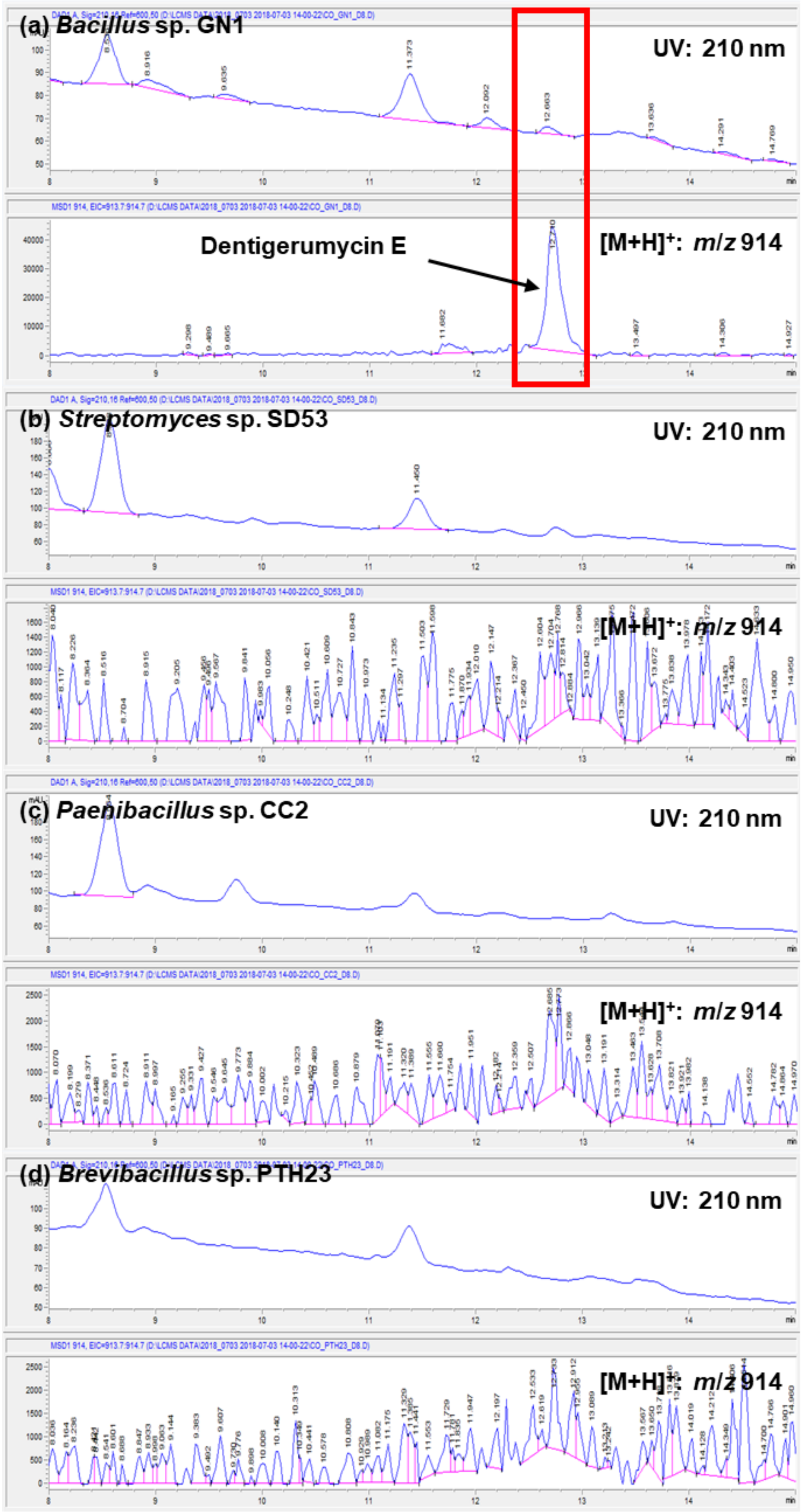

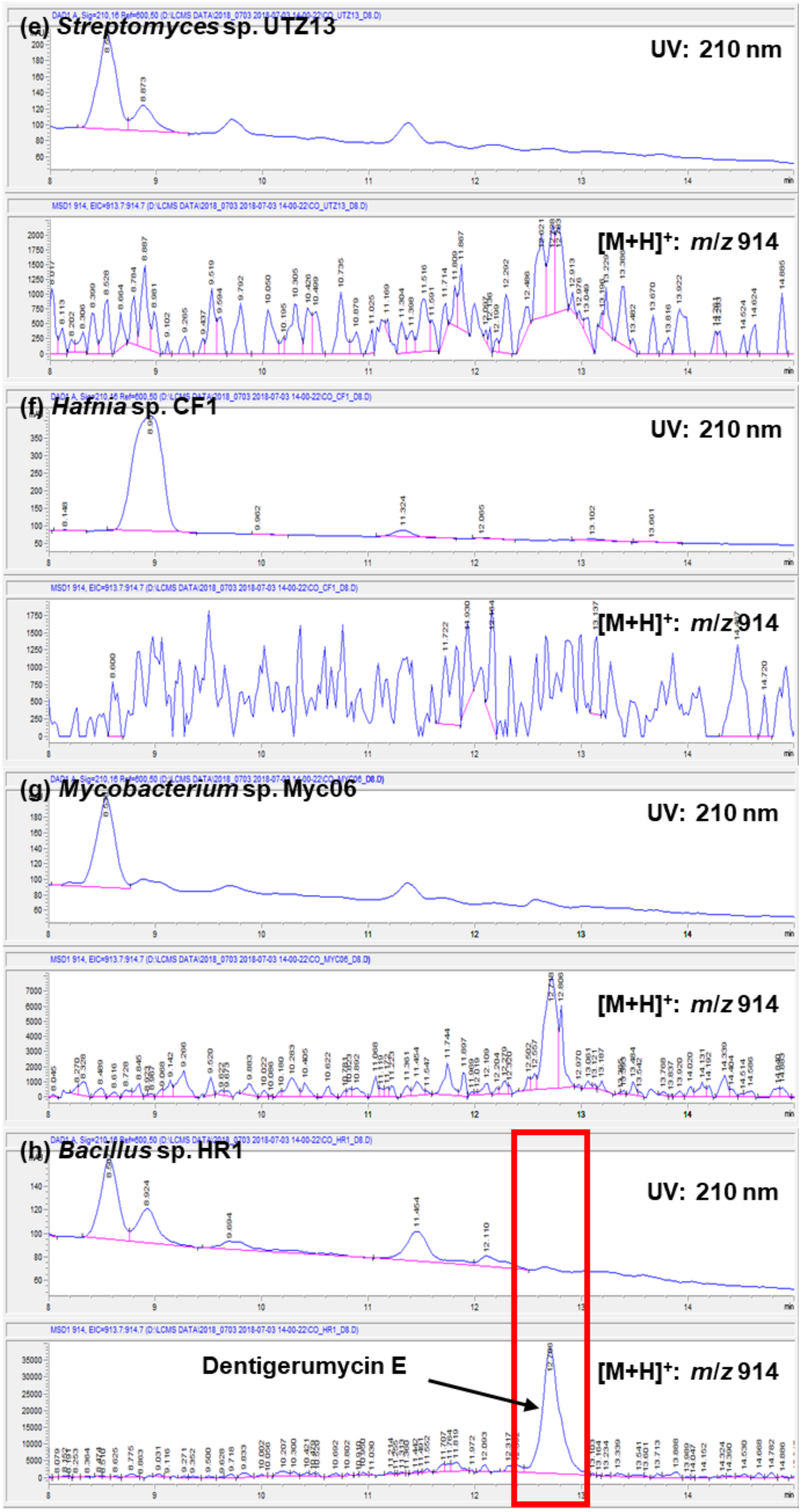
(e) Streptomyces sp. UTZ13
UV: 210 nm
[M+H]+: m/z 914
(f) Hafnia sp. CF1
UV: 210 nm
[M+H]+: m/z 914
(g) Mycobacterium sp. Myc06
UV: 210 nm
[M+H]+: m/z 914
(h) Bacillus sp. HR1
UV: 210 nm
Dentigerumycin E
[M+H]+: m/z 914

Figure S4. ^{1}H NMR spectrum (800 MHz) of dentigerumycin E (**1**) in pyridine-d_5.

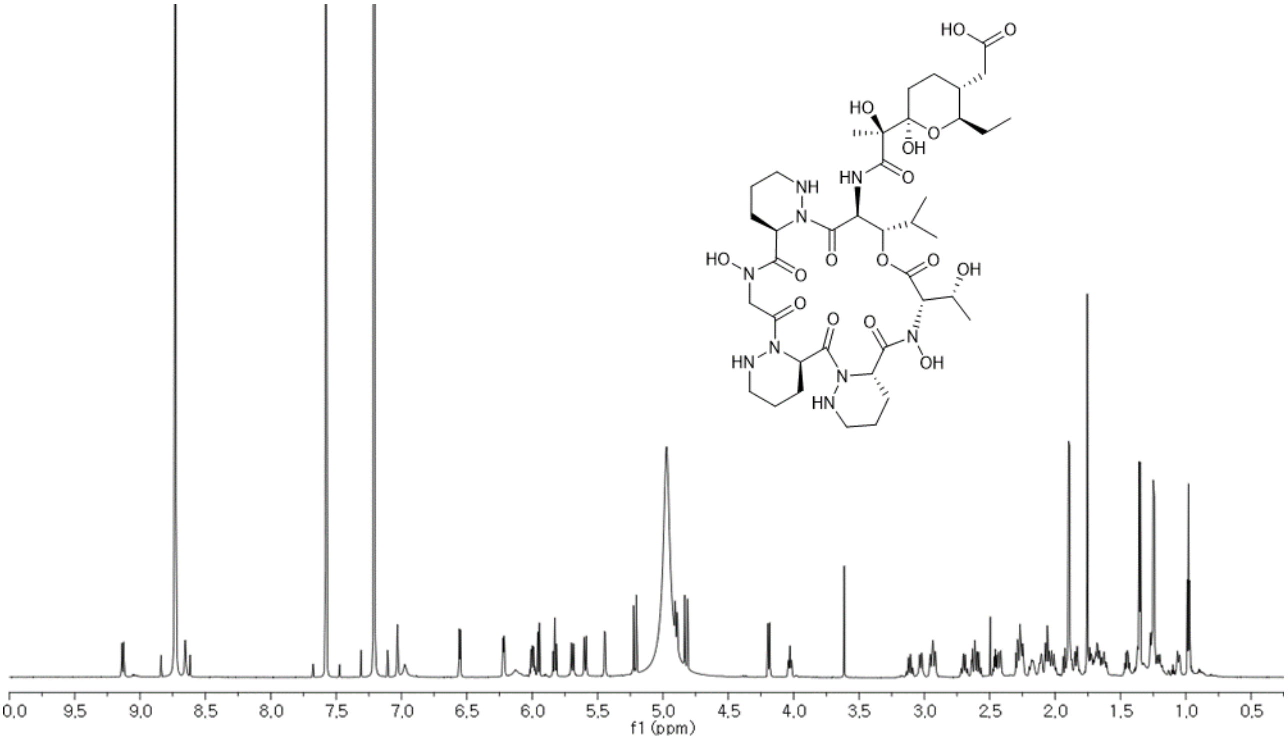

Figure S5. ^{13}C NMR spectrum (200 MHz) of dentigerumycin E (**1**) in pyridine-d_5.

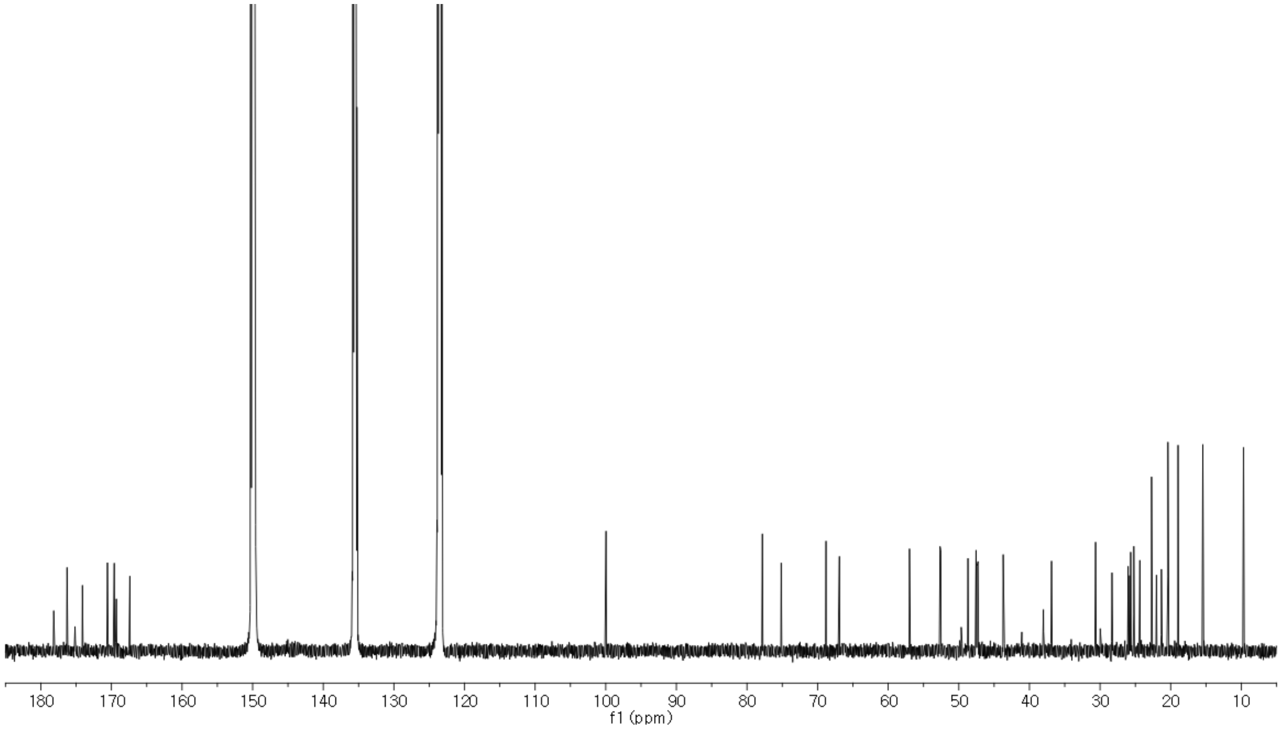

Figure S6. COSY NMR spectrum (800 MHz) of dentigerumycin E (**1**) in pyridine-d_5.

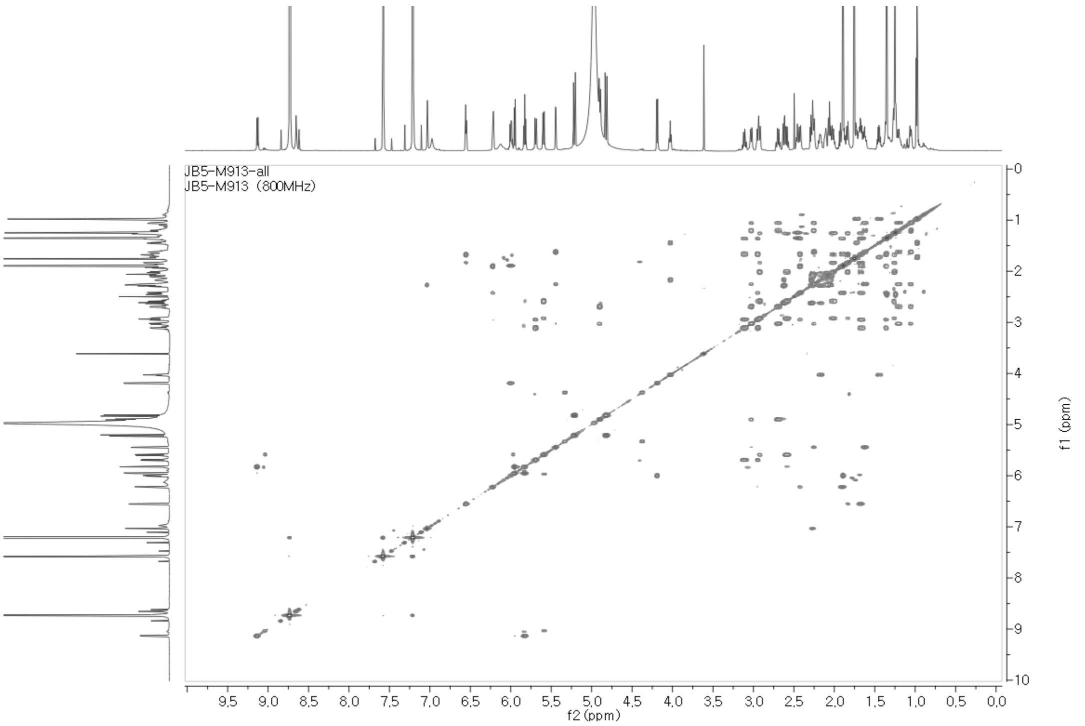

Figure S7. HSQC NMR spectrum (800 MHz) of dentigerumycin E (**1**) in pyridine-d_5.

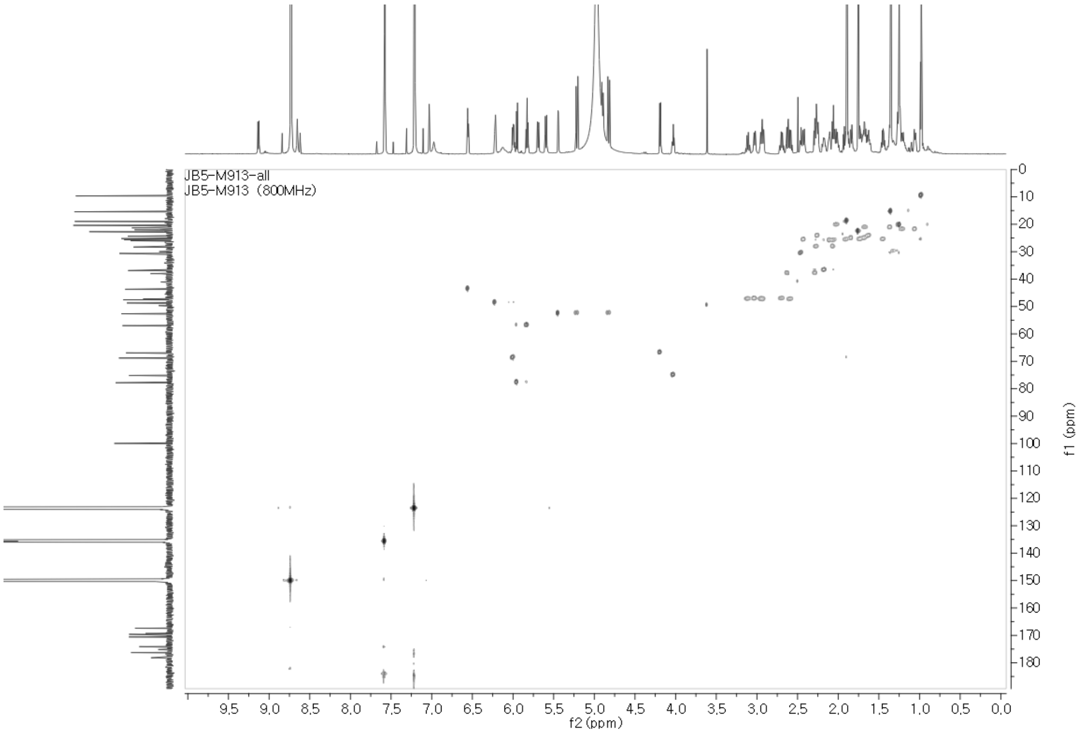

Figure S8. HMBC NMR spectrum (800 MHz) of dentigerumycin E (**1**) in pyridine-d_5.

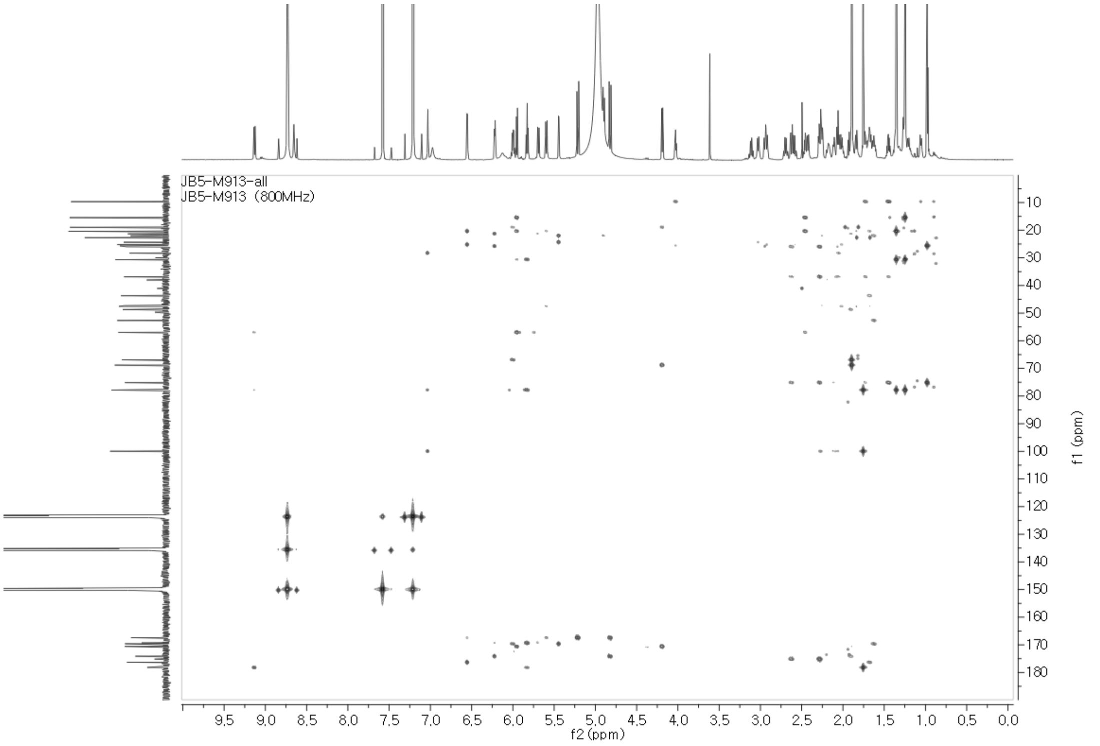

Figure S9. ROESY NMR spectrum (800 MHz) of dentigerumycin E (**1**) in Pyridine-d_5.

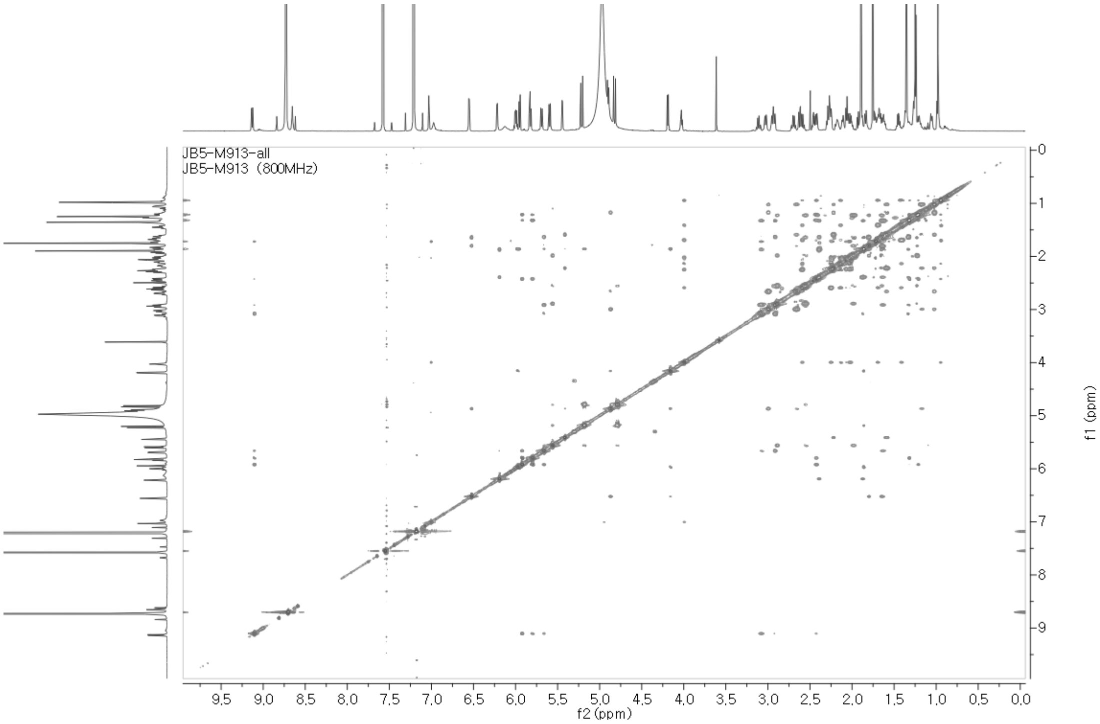

Figure S10. TOCSY NMR spectrum (800 MHz) of dentigerumycin E (**1**) in Pyridine-d_5.

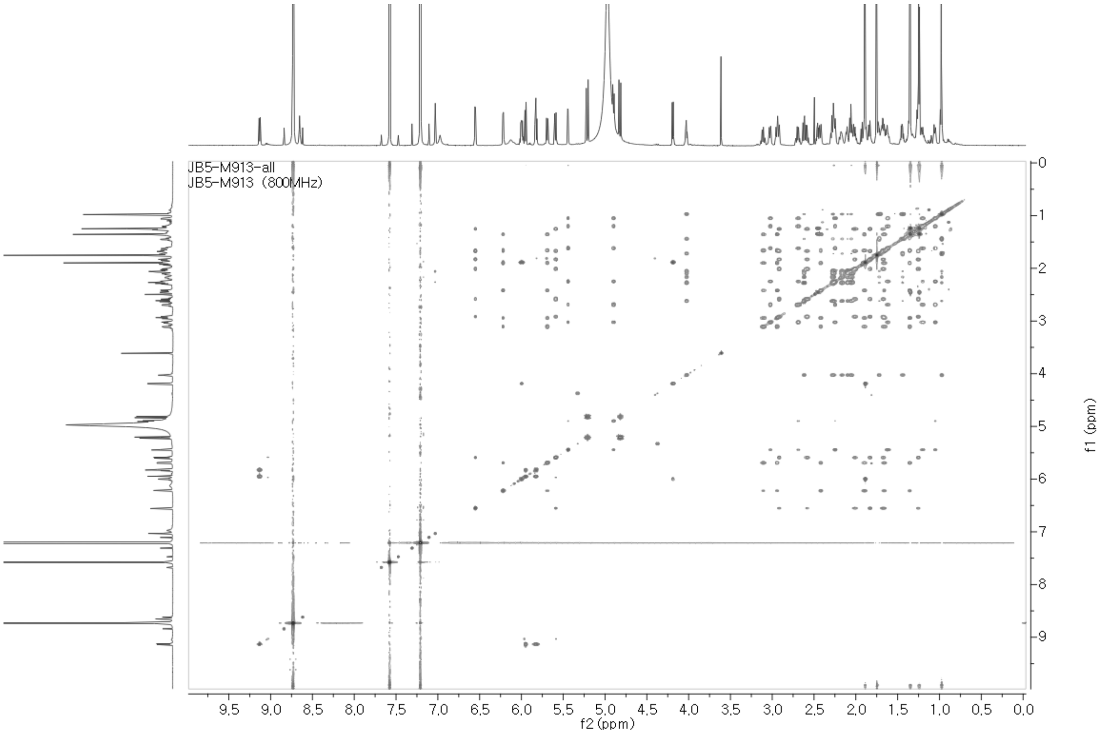

Figure S11. ^{1}H NMR spectrum (800 MHz) of 2-*N*,16-*N*-deoxydentigerumycin E (**2**) in pyridine-d_5.

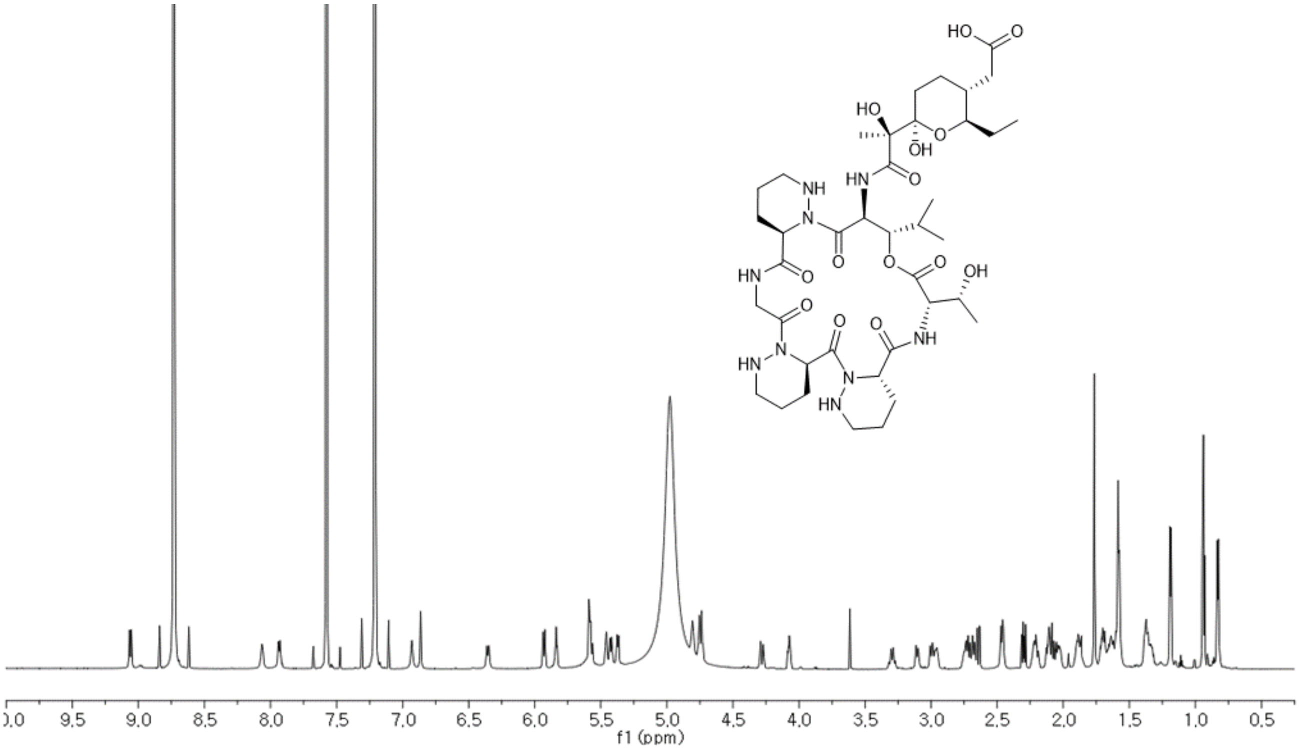

Figure S12. COSY NMR spectrum (800 MHz) of 2-*N*,16-*N*-deoxydentigerumycin E (**2**) in pyridine-d_5.

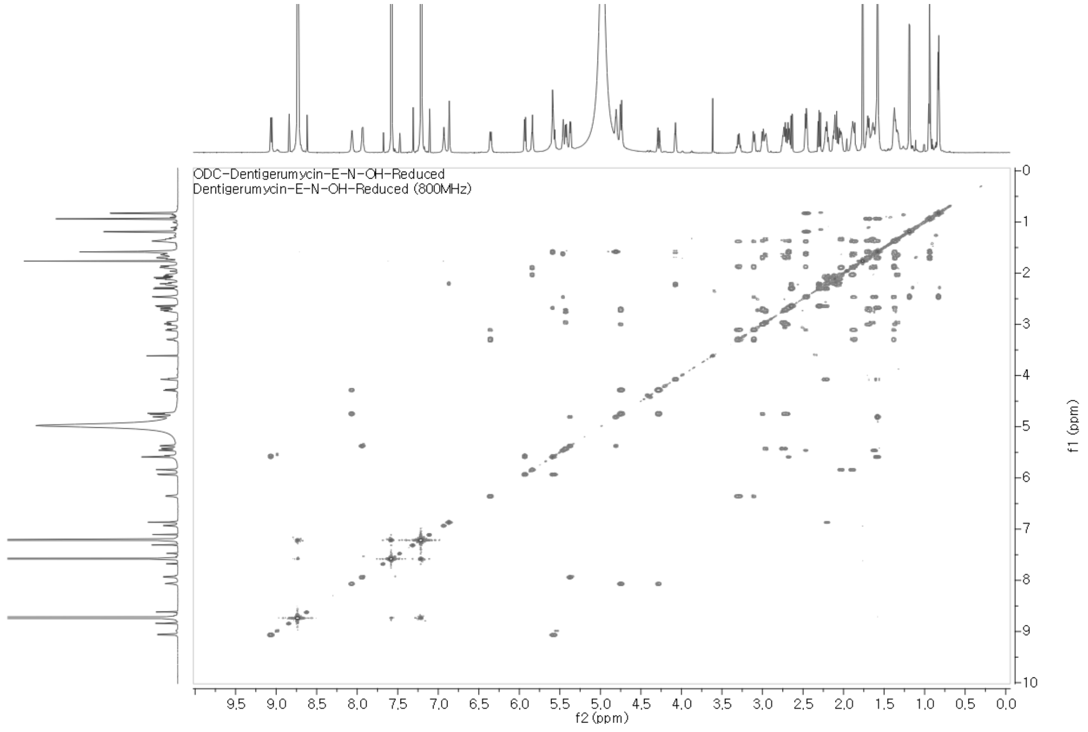

Figure S13. HSQC NMR spectrum (800 MHz) of 2-*N*,16-*N*-deoxydentigerumycin E (**2**) in pyridine-d_5.

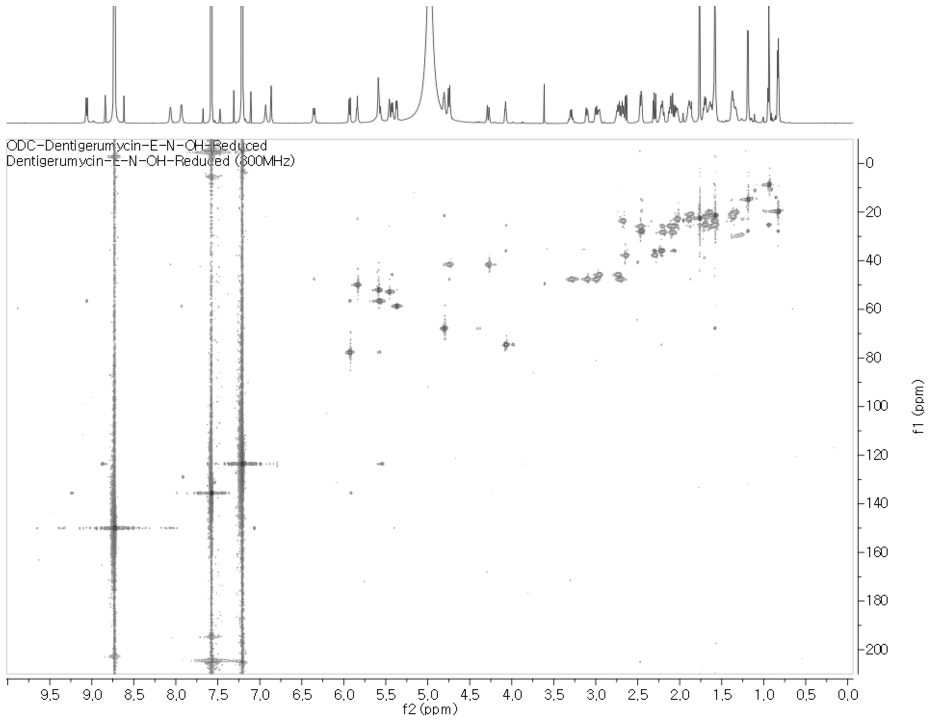

Figure S14. HMBC NMR spectrum (800 MHz) of 2-*N*,16-*N*-deoxydentigerumycin E (**2**) in pyridine-d_5.

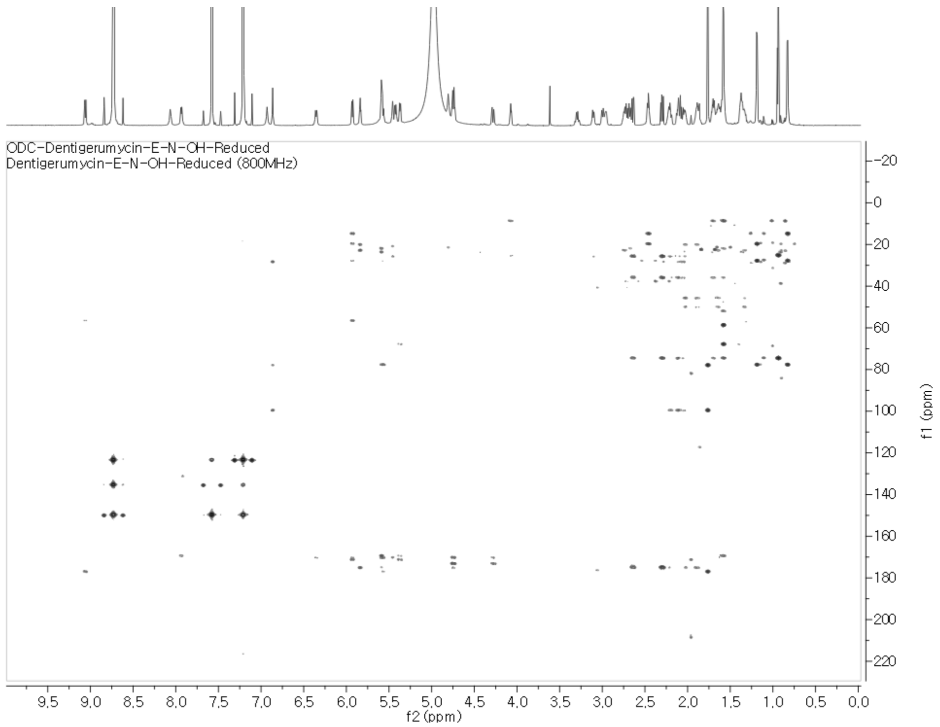

Figure S15. ROESY NMR spectrum (800 MHz) of 2-*N*,16-*N*-deoxydentigerumycin E (**2**) in pyridine-d_5.

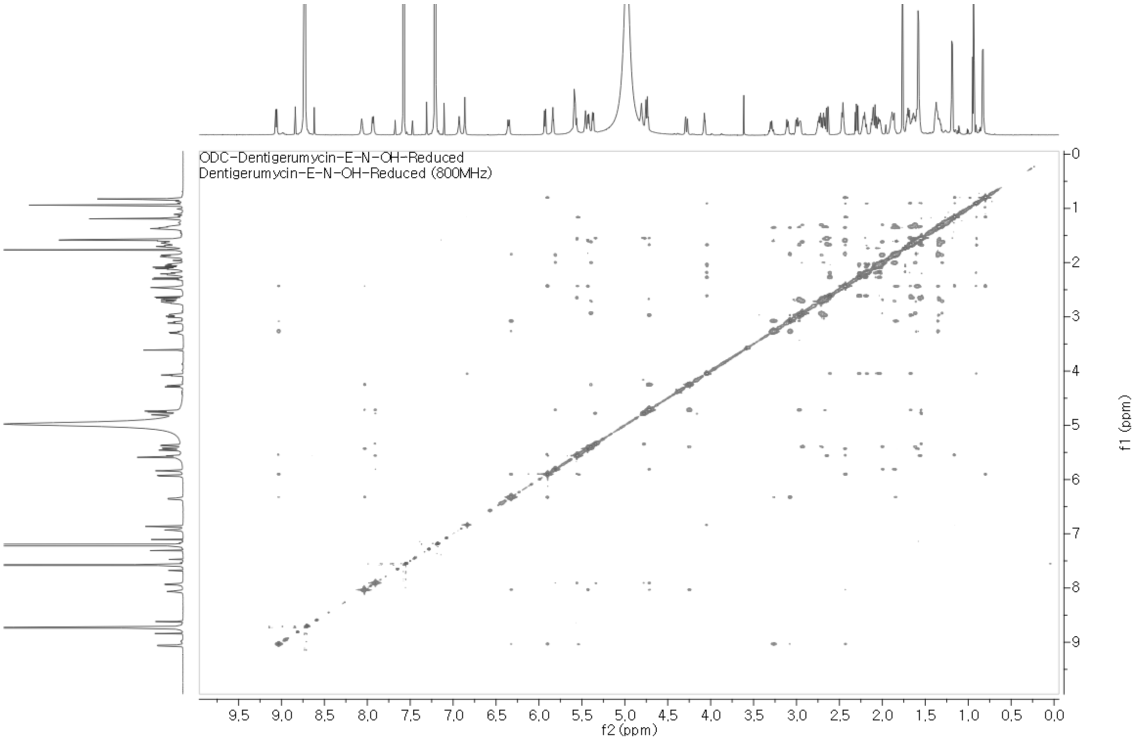

Figure S16. TOCSY NMR spectrum (800 MHz) of 2-*N*,16-*N*-deoxydentigerumycin E (**2**) in pyridine-d_5.

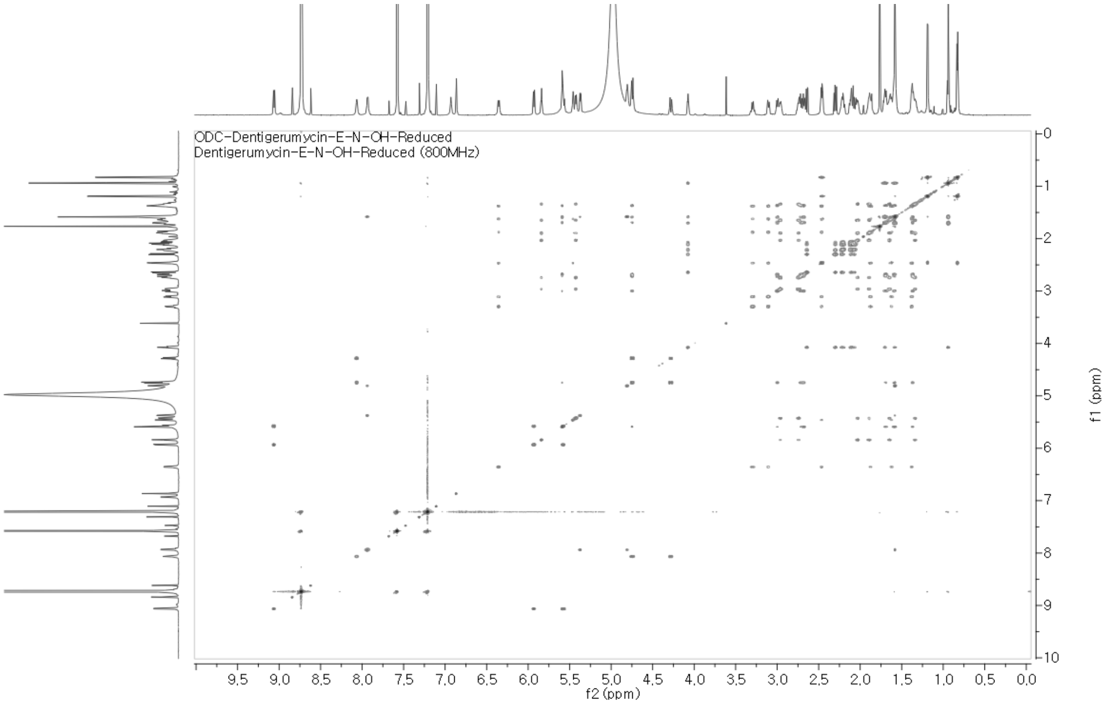

Figure S17. ^{1}H NMR spectrum (600 MHz) of dentigerumycin E methyl ester (**3**) in pyridine-d_5.

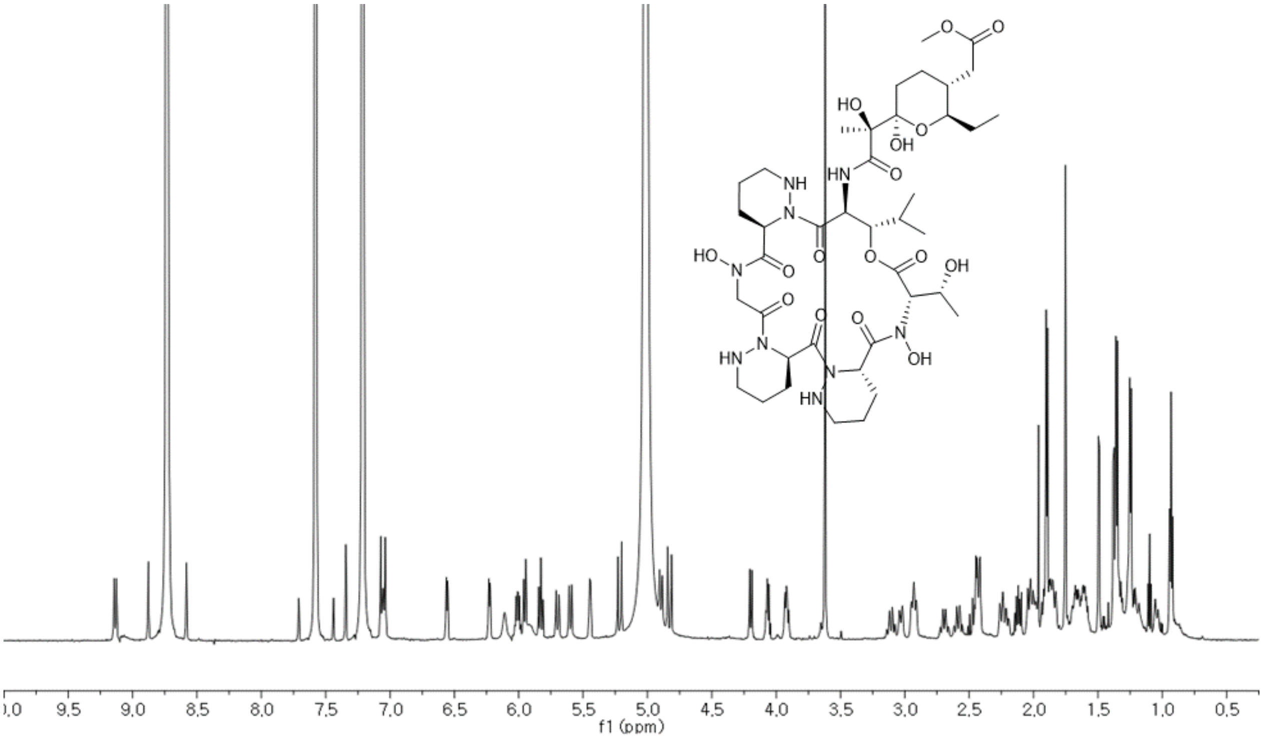

Figure S18. COSY NMR spectrum (600 MHz) of dentigerumycin E methyl ester (**3**) in pyridine-d_5.

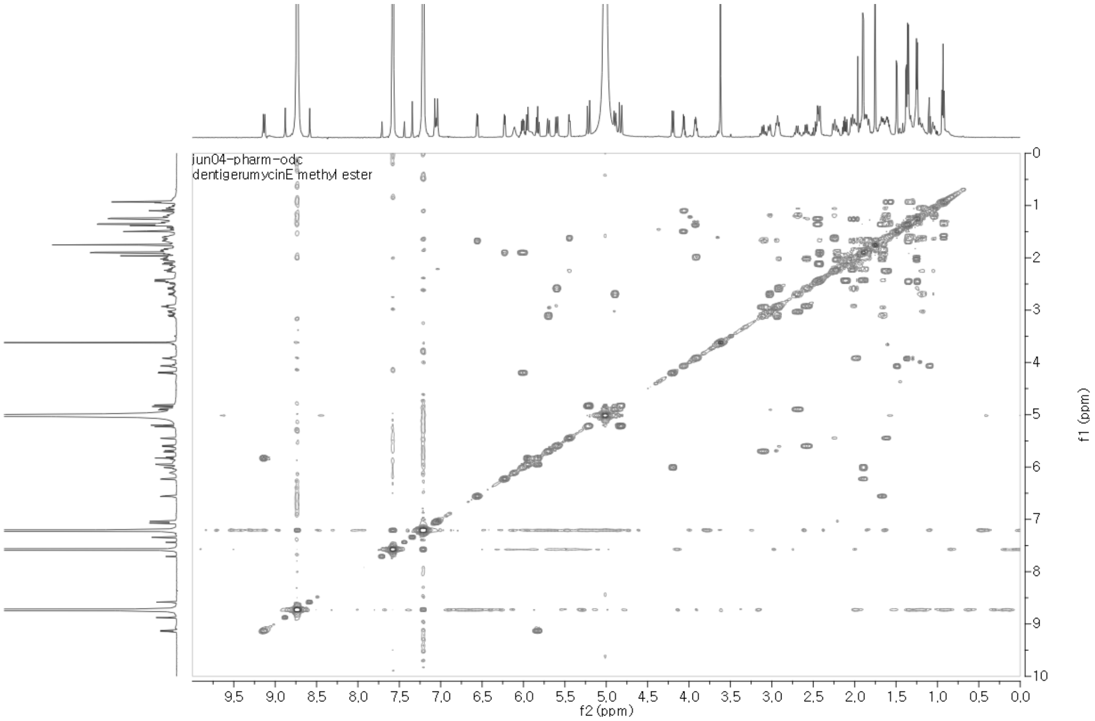

Figure S19. HSQC NMR spectrum (600 MHz) of dentigerumycin E methyl ester (**3**) in pyridine-d_5.

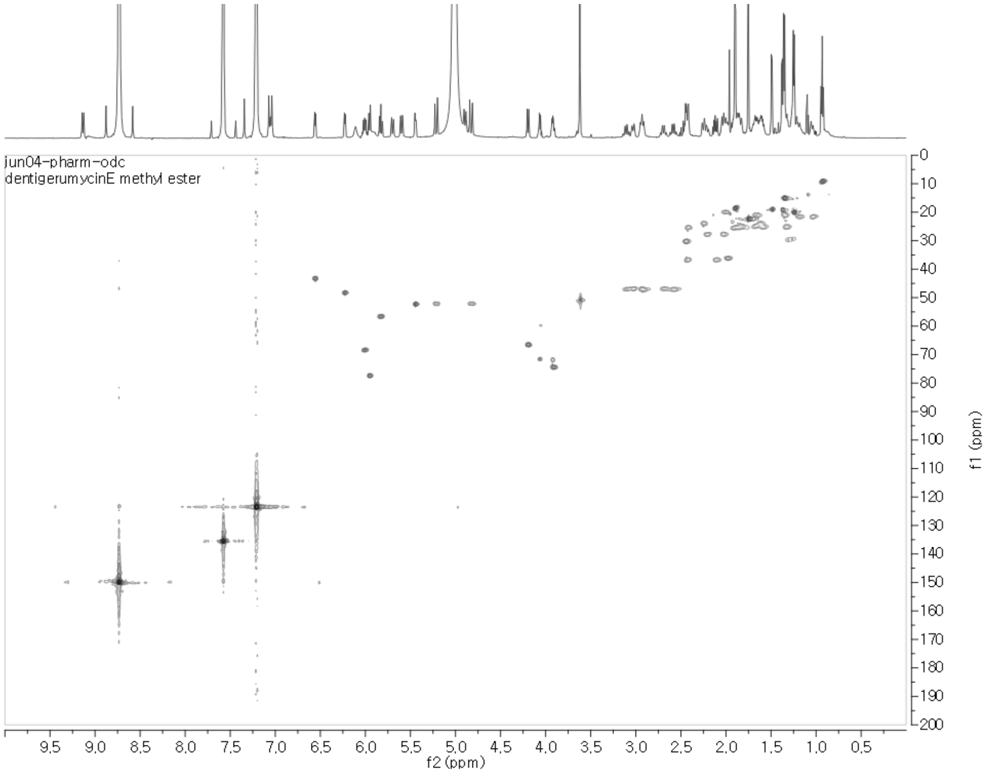

Figure S20. HMBC NMR spectrum (600 MHz) of dentigerumycin E methyl ester (**3**) in pyridine-d_5.

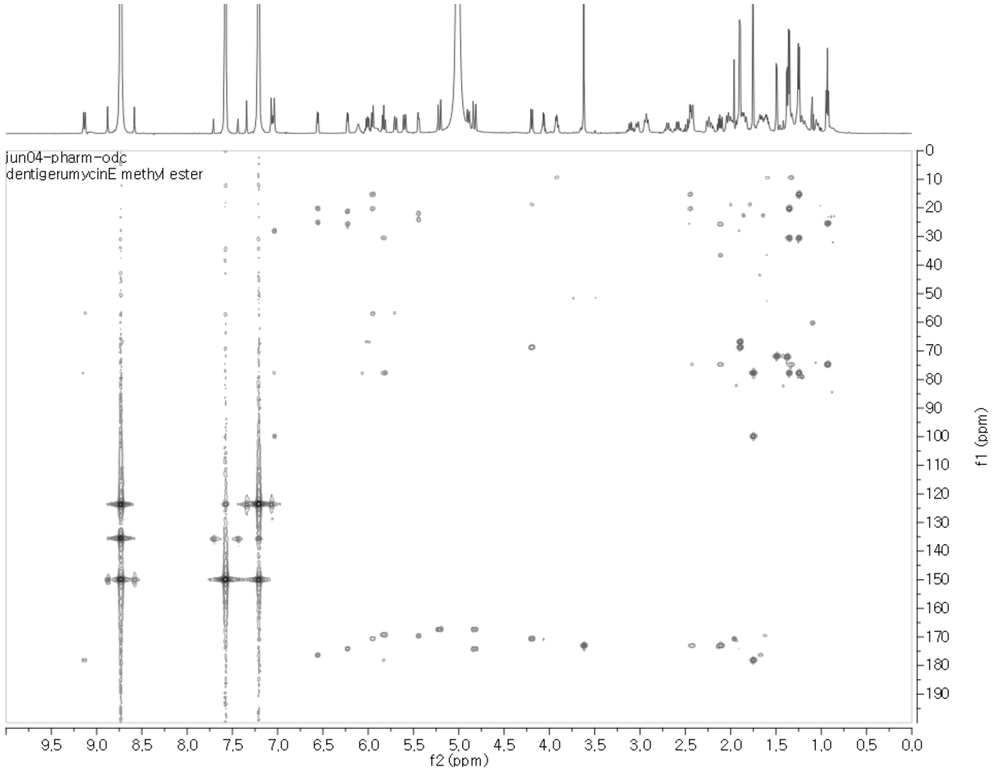

Figure S21. ROESY NMR spectrum (600 MHz) of dentigerumycin E methyl ester (**3**) in pyridine-d_5.

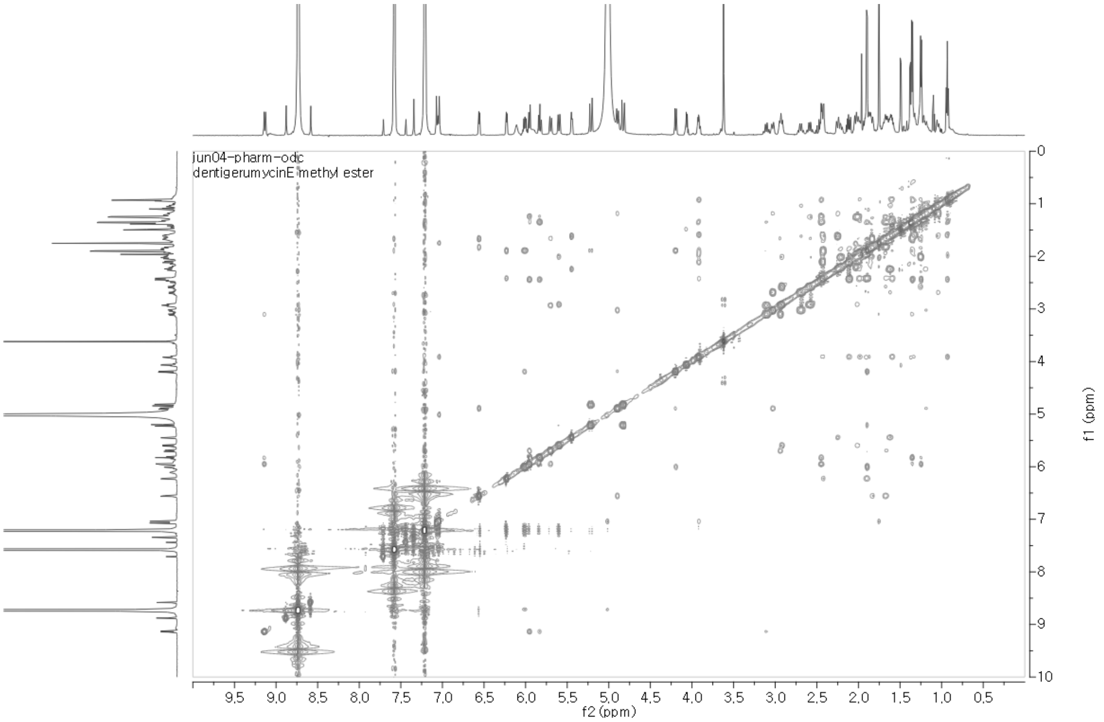

Figure S22. TOCSY NMR spectrum (600 MHz) of dentigerumycin E methyl ester (**3**) in pyridine-d_5.

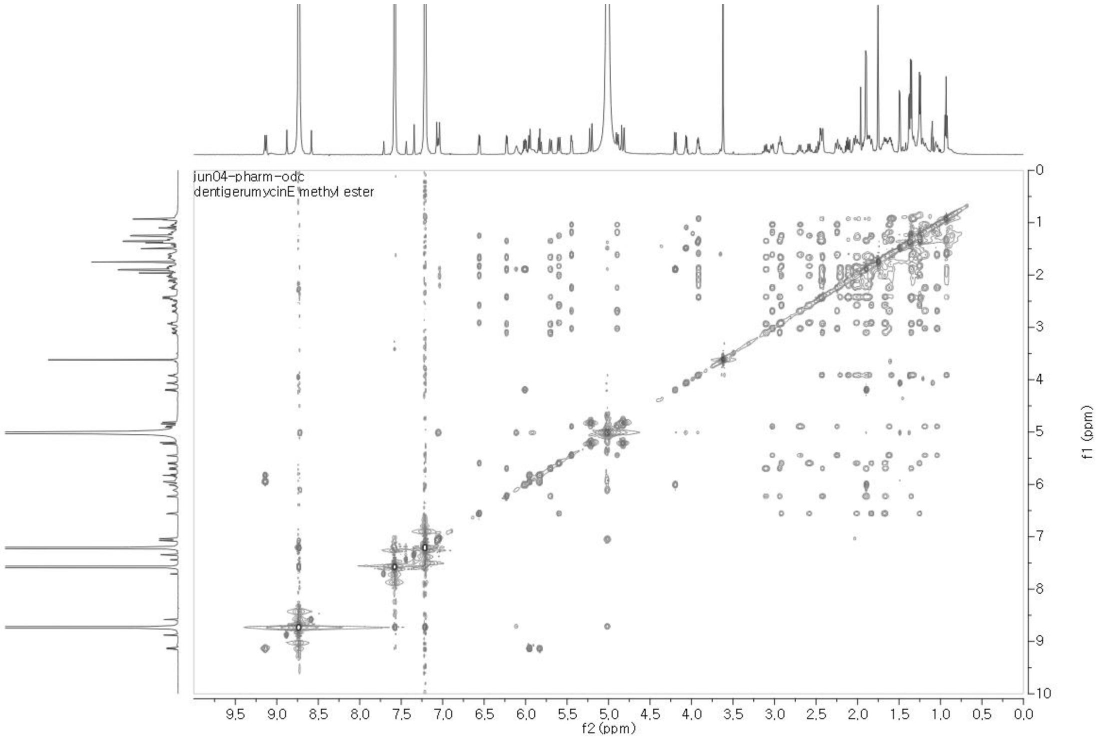

Table S1. NMR spectral data for 2-*N*,16-*N*-deoxydentigerumycin (**2**) and dentigerumycin E methyl ester (**3**) in pyridine-d_5.

Position	**2**[a]		**3**[b]	
	**δ$_C$, Type**	**δ$_H$, mult (*J* in Hz)**	**δ$_C$, Type**	**δ$_H$, mult (*J* in Hz)**
1	172.2,		170.6, C	
2	58.7, CH	5.77, dd (9.5, 4.0)	66.6, CH	4.19, d (10.0)
2-N-OH	–	–		n.d.
2-NH		7.93, d (9.5)	–	–
3	67.8, CH	4.81, m	68.4, CH	6.01, m
3-OH		n.d.		n.d.
4	21.4, CH_3	1.58, d (6.5)	18.6, CH_3	1.90, d (6.5)
5	169.4, C		169.6, C	
6	52.0, CH	5.59, m	52.4, CH	5.45, dd (5.5, 1.5)
7a	23.6, CH_2	2.68, br. d (13.5)	24.0, CH_2	2.24, br. d (14.5)
7b		1.59, m		1.61, m
8a	21.9, CH_2	1.69, m	21.6, CH_2	1.19, m
8b		1.36, m		1.06, br. d (13.0)
9a	47.7, CH_2	3.00, br. d (13.0)	46.9, CH_2	3.03, br. d (13.0)
9b		2.71, m		2.69, m
9-NH		4.75, br. d (12.5)		4.90, br. d (13.0)
10	175.1, C		176.3, C	
11	49.9, CH	5.84, dd (6.0, 6.0)	43.3, CH	6.56, br. d (6.5)
12a	22.9, CH_2	2.03, m	25.0, CH_2	1.84, br. d (13.0)
12b		1.89, m		1.68, m
13a	20.1, CH_2	1.64, m	20.0, CH_2	2.01, br. d (13.0)
13b		1.33, m		1.27, m
14a	45.7, CH_2	2.96, m	47.2, CH_2	2.93, br. d (12.5)
14b		2.74, m		2.59, m
14-NH		5.42, dd (11.5, 3.0)		5.60, dd (12.5, 1.0)
15	173.1, C		167.4, C	
16a	41.5, CH_2	4.74, m	52.2, CH_2	5.21, d (17.5)
16b		4.28, dd (18.5, 3.5)		4.83, d (17.5)
16-N-OH	–	–		n.d.
16-NH		8.07, br. s	–	–
17	170.2, C		174.2, C	
18	52.8, CH	5.46, br. s	48.3, CH	6.23, br. d (6.5)
19a	25.9, CH_2	2.46, m	25.5, CH_2	2.42, br. d (13.0)
19b		1.62, m		1.91, m

20a	20.9, CH_2	1.87, m	21.0, CH_2	1.65, m
20b		1.38, m		1.34, m
21a	47.65, CH_2	3.29, m	47.0, CH_2	3.10, m
21b		3.10, br. d (13.0)		2.93, br. d (12.5)
21-NH		6.35, br. d (12.0)		5.70, br. d (12.5)
22	170.3, C		169.3, C	
23	56.6, CH	5.58, br. d (11.0)	56.6, CH	5.83, dd (10.5, 10.0)
23-NH		9.06, d (11.0)		9.13, d (10.5)
24	77.7, CH	5.93, br. d (11.0)	77.5, CH	5.95, dd (10.0, 1.0)
25	27.8, CH	2.46, m	30.3, CH	2.46, m
26	14.8, CH_3	1.19, d (7.0)	15.1, CH_3	1.35, d (7.0)
27	19.7, CH_3	0.83, d (7.0)	20.1, CH_3	1.25, d (7.0)
28	176.9, C		178.1, C	
29	77.9, C		77.8, C	
29-OH		6.93, s		7.05, s
30	99.6, C		99.9, C	
30-OH		6.86, s		7.03, s
31a	28.4, CH_2	2.19, m	27.9, CH_2	2.20, m
31b		2.09, m		2.02, m
32a	25.7, CH_2	2.11, m	25.7, CH_2	1.91, m
32b		2.06, m		1.86, m
33	35.9, CH	2.21, m	36.6, CH	1.98, m
34	74.6, CH	4.07, m	74.8, CH	3.92, m
35	22.5, CH_3	1.76, s	22.7, CH_3	1.75, s
36a	37.8, CH_2	2.64, dd (15.0, 4.5)	37.1, CH_2	2.43, dd (15.0, 4.5)
36b		2.30, dd (15.0, 9.0)		2.11, dd (15.0, 8.5)
37	174.9, C		173.0,	
37-OH		n.d.	–	–
38	–	–	52.4, CH_3	3.62, s
38a	25.2, CH_2	1.70, m	–	–
38b		1.57, m	–	–
39	8.7, CH_3	0.94, t (7.5)	–	–
39a	–	–	25.5, CH_2	1.60, m
39b	–	–		1.34, m
40	–	–	9.5, CH_3	0.93, t (7.5)

[a] ^{1}H 800 MHz, ^{13}C 200 MHz. [b] ^{1}H 600 MHz, ^{13}C 125 MHz.

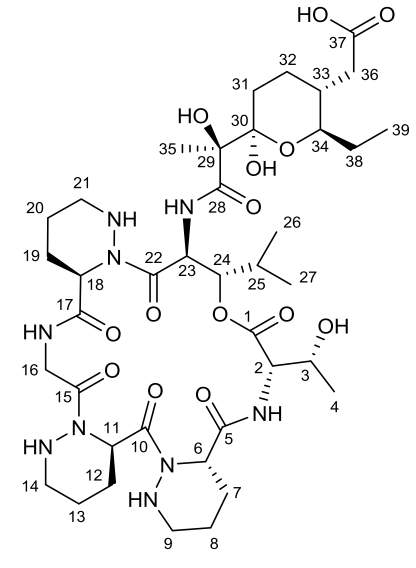

2

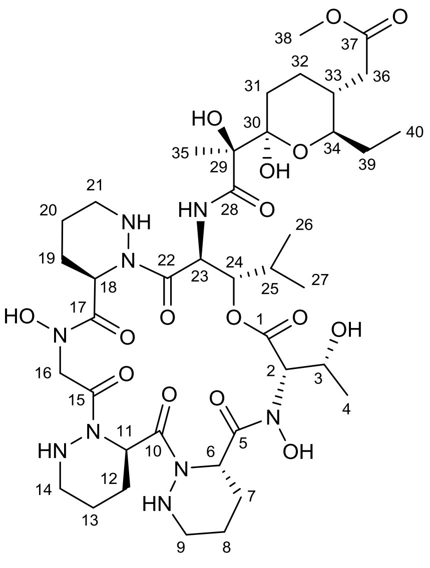

3

Figure S23. LC/MS chromatogram of (a) L- and (b) D-FDAA derivatives of **1**, and (c) L- and D-FDAA derivatives of threonine in **2**.

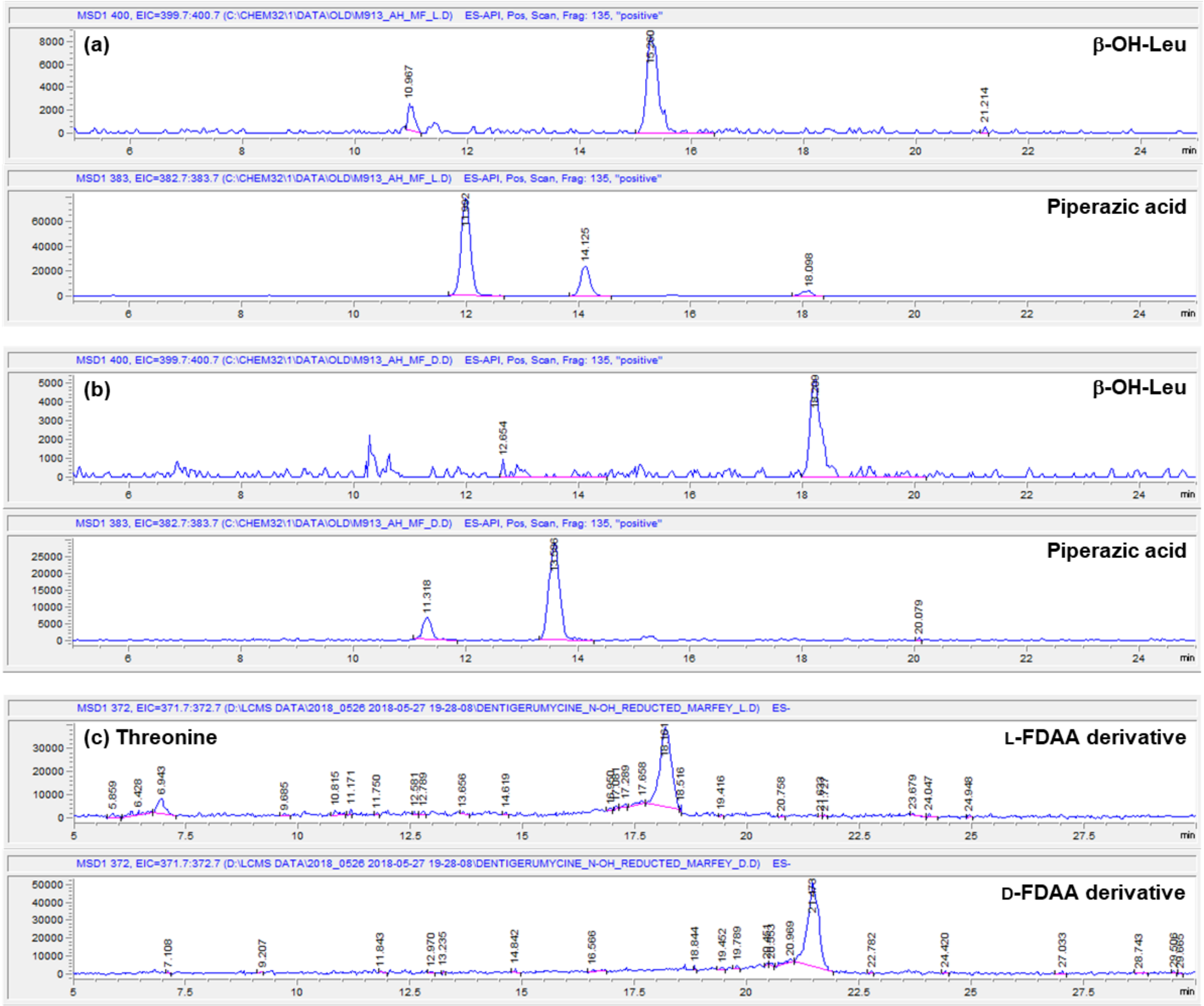

Figure S24. LC/MS chromatogram of GITC of (a) **2**, (b) authentic L-Thr, and (c) authentic L-*allo*-Thr.

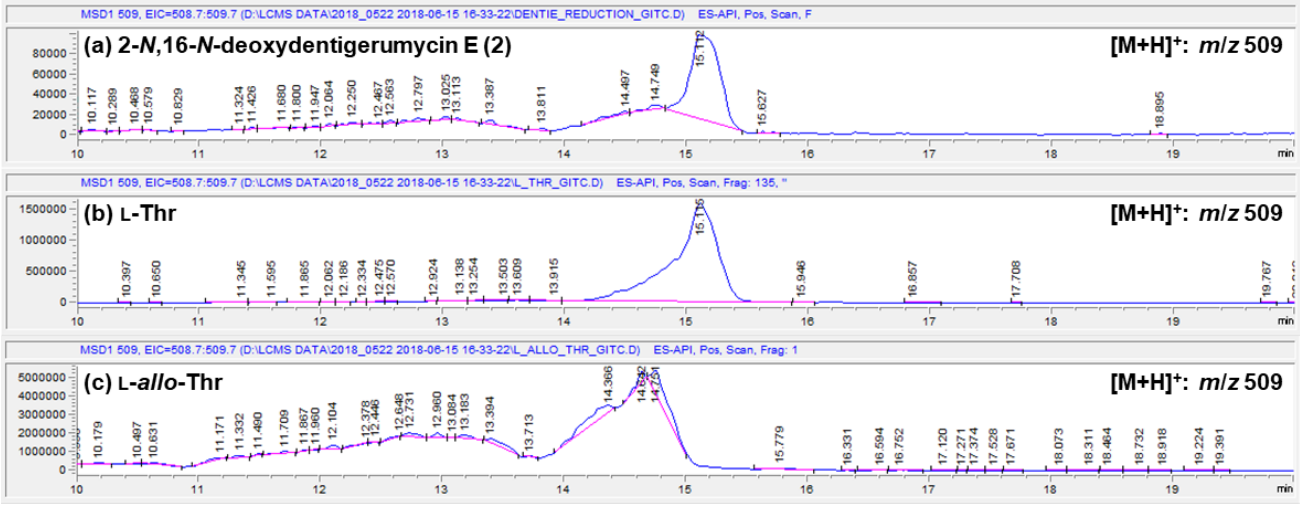

Table S2. Alignment of the homologous proteins from the dentigerumycin E biosynthetic gene cluster.

Gene	Deduced function of the encoded protein	aa
6583	Methylmalonyl-CoA mutase	754
6584	Thioesterase	252
6585	Unknown	425
6586	Acylamino-acid-releasing enzyme	649
6587	Unknown	60
6588	Putative cytochrome P450 hydroxylase	435
6589	3-oxoacyl synthase	329
6590	Non-ribosomal peptide synthetase	1032
6591	Malonyl CoA-acyl carrier protein transacylase	1050
6592	Malonyl CoA-acyl carrier protein transacylase	2157
6593	Malonyl CoA-acyl carrier protein transacylase	1852
6594	Malonyl CoA-acyl carrier protein transacylase	1029
6595	Thioesterase	245
6596	Putative cytochrome P450 hydroxylase	416
6597	Pyoverdine sidechain non-ribosomal peptide	89
6598	3-(3-hydroxy-phenyl) propionate hydroxylase	500
6599	Unknown	172
6600	Unknown	280
6601	Putative cytochrome P450 hydroxylase	408
6602	Unknown	171
6603	Thioesterase	253
6604	Non-ribosomal peptide synthetase	1293
6605	Non-ribosomal peptide synthetase	2587
6606	Non-ribosomal peptide synthetase	2593
6607	Unknown	395
6608	Pyoverdine sidechain non-ribosomal peptide	77
6609	Non-ribosomal peptide synthetase	526
6610	Unknown	244
6611	Polymyxin synthetase	79
6612	Malonyl CoA-acyl carrier protein transacylase	248
6613	D-alanyl-D-alanine carboxypeptidase	386
6614	N-acetyl-gamma-glutamyl phosphate reductase	342
6615	Glutamate N-acetyltransferase	383
6616	Acetylglutamate kinase	307
6617	Acetylornithine aminotransferase	405
6618	Unknown	241
6619	Trascription regulator	214
6620	L-ornithin 5-monooxygenase	450

Figure S25. Sequence alignment of KS domains from BGC of dentigerumycin E. Yellow boxes indicate conserved motifs of KS domains and red box indicates the replacement of the active site cysteine (C) to glutamine (Q).

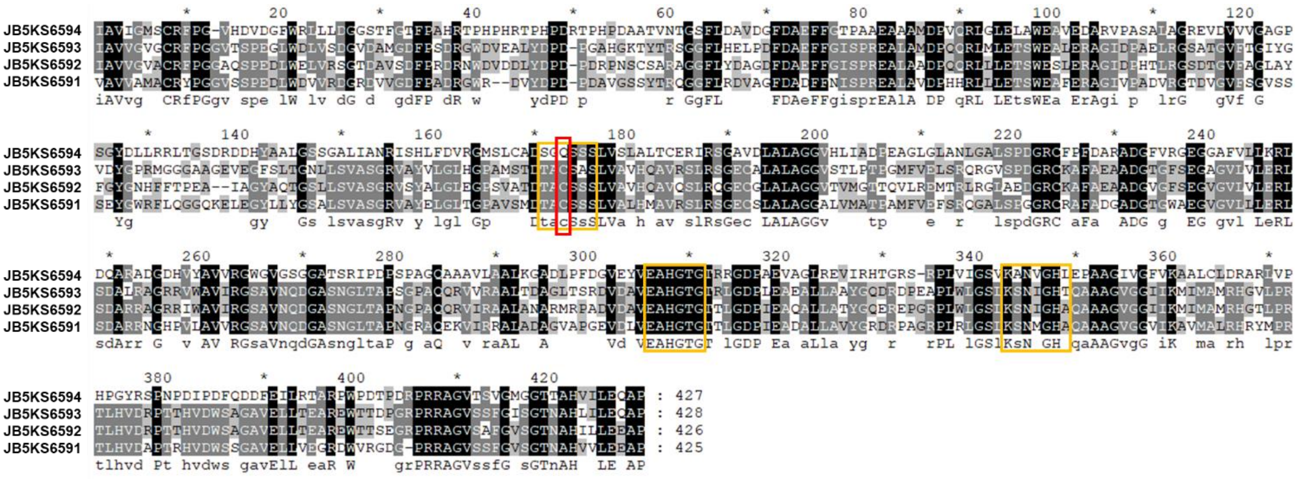

Figure S26. Sequence alignment of DH domains from BGC of dentigerumycin E and other PKSs (Du, Y., Wang, Y., Huang, T., Tao, M., Deng, Z., and Lin, S. (2014). Identification and characterization of the biosynthetic gene cluster of polyoxypeptin A, a potent apoptosis inducer. *BMC Microbiol*. 14, 30. doi: 10.1186/1471-2180-14-30).

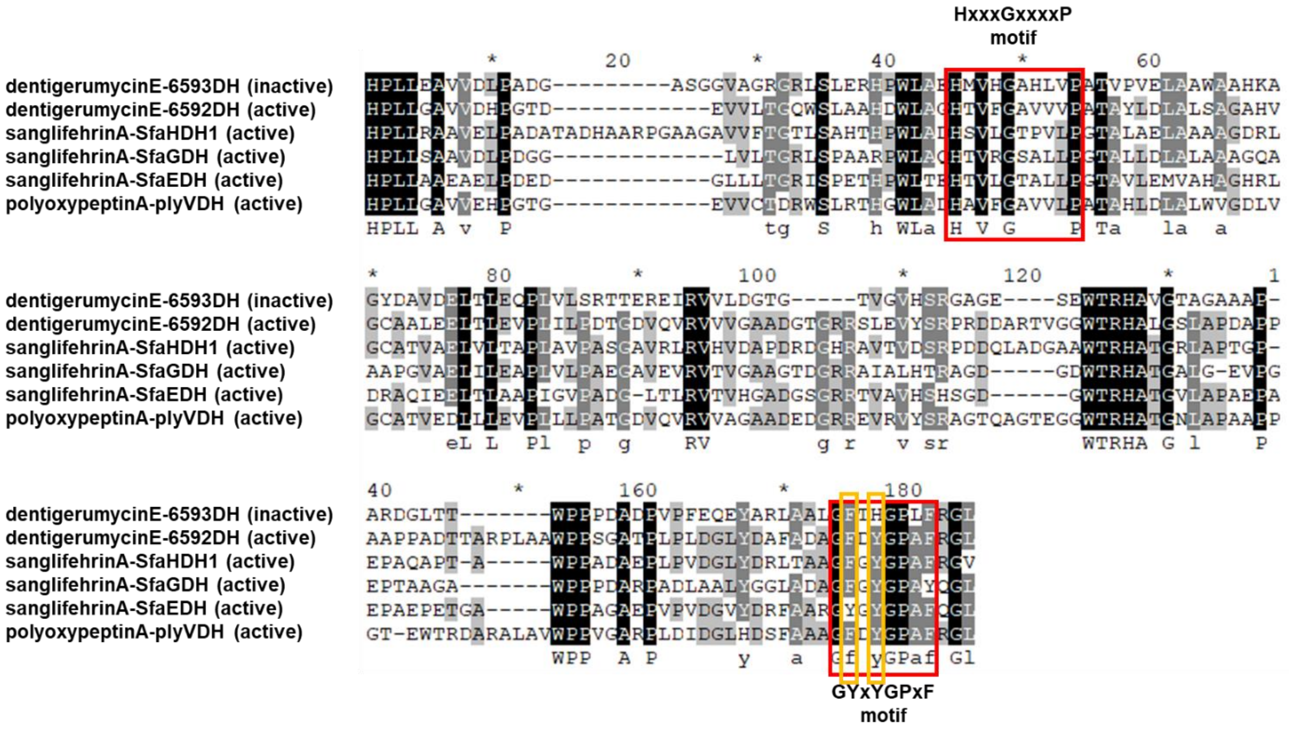

Table S3. Antiproliferative activities of **1-3** against various human cancer cell lines and normal breast epithelial cells.

**Compound**	**Observed IC_{50} (μM)**					
	**A549**	**HCT116**	**MDA-MB-231**	**SK-HEP-1**	**SNU638**	**MCF-10A**
**Dentigerumycin E (1)**	38	28	28	27	39	>50
**2-*N*,16-*N*-deoxydentigerumycin E (2)**	>50	>50	>50	>50	>50	>50
**Dentigerumycin E methyl ester (3)**	>50	>50	>50	>50	>50	>50
**Etoposide**	0.42	0.66	6.21	0.42	0.57	>20

Figure S27. Wound healing assay of 2-*N*,16-*N*-deoxydentigerumycin E (**2**) and dentigerumycin E methyl ester (**3**).

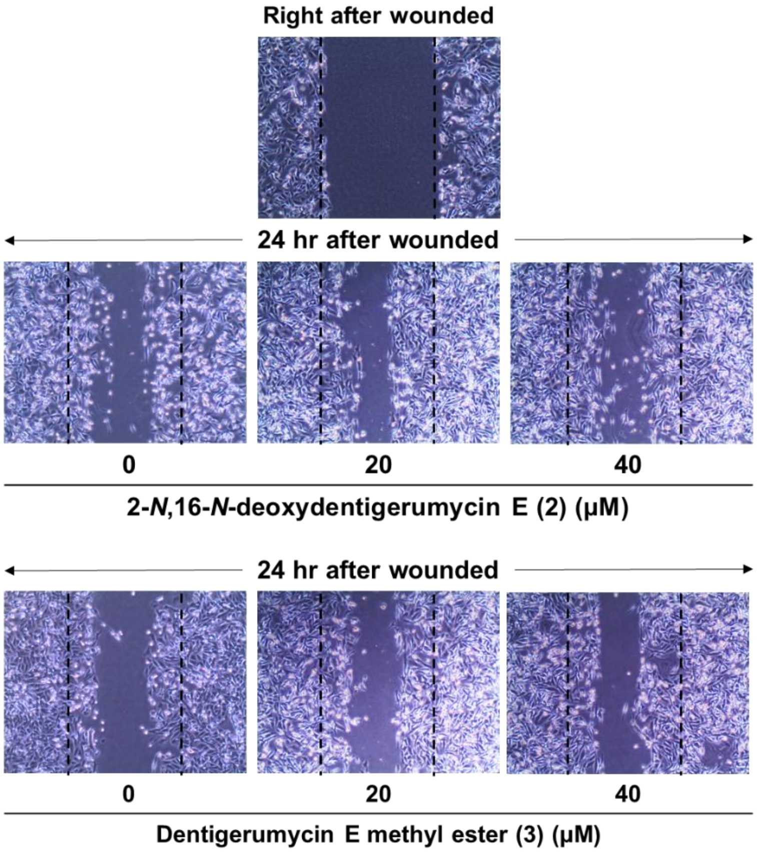

Figure S28. Transwell cell invasion assay of 2-*N*,16-*N*-deoxydentigerumycin E (**2**) and dentigerumycin E methyl ester (**3**).

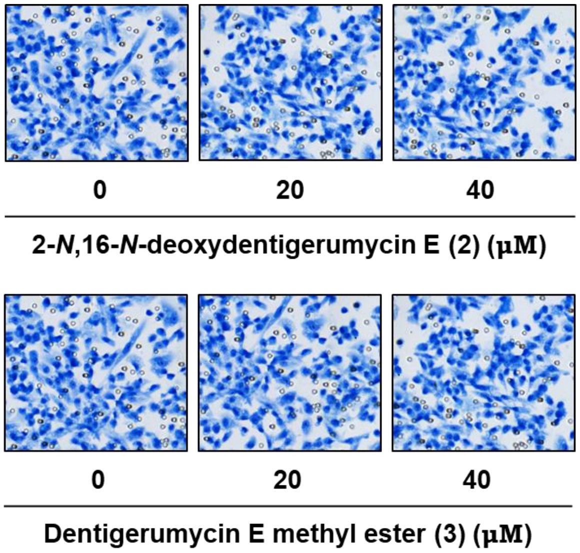

Supplement: Supplementary file 1 [file Data_Sheet_1.PDF]
